# SARS-CoV-2 envelope protein alters calcium signaling via SERCA interactions

**DOI:** 10.1038/s41598-024-71144-5

**Published:** 2024-09-11

**Authors:** Blanka Berta, Hedvig Tordai, Gergely L. Lukács, Béla Papp, Ágnes Enyedi, Rita Padányi, Tamás Hegedűs

**Affiliations:** 1 Institute of Biophysics and Radiation Biology, Semmelweis University, Budapest, Hungary; 2 Department of Physiology, McGill University, Montréal, QC Canada; 3 INSERM UMR U976, Hôpital Saint-Louis, Paris, France; 4 Institut de Recherche Saint-Louis, Hôpital Saint-Louis, Université de Paris, Paris, France; 5 CEA, DRF-Institut Francois Jacob, Department of Hemato-Immunology Research, Hôpital Saint-Louis, Paris, France; 6 Department of Transfusiology, Semmelweis University, Budapest, Hungary; 7 HUN-REN-SU Biophysical Virology Research Group, Eötvös Loránd Research Network, Budapest, Hungary

**Keywords:** SERCA, COVID-19, SARS-CoV-2, Envelope protein, Ca^2+^ signaling, Regulin, Calcium signalling, SARS-CoV-2

## Abstract

The clinical management of severe COVID-19 cases is not yet well resolved. Therefore, it is important to identify and characterize cell signaling pathways involved in virus pathogenesis that can be targeted therapeutically. Envelope (E) protein is a structural protein of the virus, which is known to be highly expressed in the infected host cell and is a key virulence factor; however, its role is poorly characterized. The E protein is a single-pass transmembrane protein that can assemble into a pentamer forming a viroporin, perturbing Ca^2+^ homeostasis. Because it is structurally similar to regulins such as, for example, phospholamban, that regulate the sarco/endoplasmic reticulum calcium ATPases (SERCA), we investigated whether the SARS-CoV-2 E protein affects the SERCA system as an exoregulin. Using FRET experiments we demonstrate that E protein can form oligomers with regulins, and thus can alter the monomer/multimer regulin ratio and consequently influence their interactions with SERCAs. We also confirm that a direct interaction between E protein and SERCA2b results in a decrease in SERCA-mediated ER Ca^2+^ reload. Structural modeling of the complexes indicates an overlapping interaction site for E protein and endogenous regulins. Our results reveal novel links in the host-virus interaction network that play an important role in viral pathogenesis and may provide a new therapeutic target for managing severe inflammatory responses induced by SARS-CoV-2.

## Introduction

The SARS-CoV-2 coronavirus causes acute respiratory distress syndrome that emerged as a major global threat with high mortality^1^. One of the four structural proteins, the envelope (E) protein plays an essential role in pathogenicity and virus replication^2^. E protein is involved in viral assembly^3^ and its deletion significantly decreases virulence^4^. This viral protein has been found to be highly expressed in infected cells during the CoV replication cycle, also indicating that this protein is crucial for controlling cellular functions during replication^5^.

**Fig. 1:**
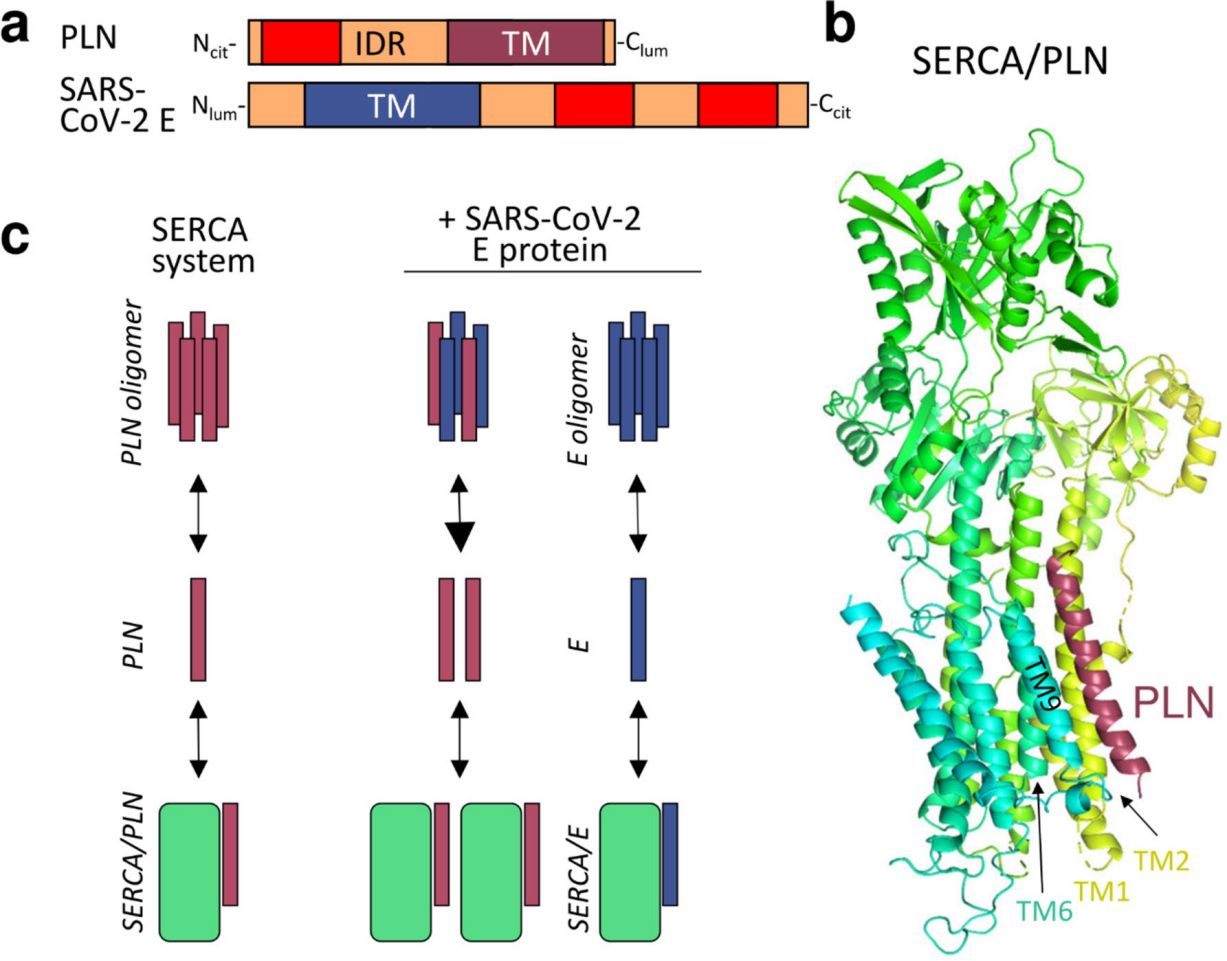
(**a**) Topology of PLN and E proteins. TM: transmembrane helix; IDR: intrinsically disordered region; red: potential MemMoRF. (**b**) The X-ray structure (PDBID: 4kyt) of the rabbit SERCA (yellow-green-turquoise)—PLN (burgundy) complex. (**c**) Left side shows the SERCA-PLN system. Right side depicts that E protein may interfere with the SERCA system by perturbing the PLN pentamer pool or by interacting with SERCA. burgundy: PLN; blue: E protein; green: SERCA.

The E protein is a small transmembrane (TM) protein of 75 amino acid (a.a.) residues with a single TM helix, consisting of a short N-terminal tail (1–8 a.a.) and a longer C-terminal segment (38–75 a.a.) (Fig. 1a). The C-terminus of E protein harbors a PDZ binding motif (PBM) that interacts with human PDZ proteins, such as PALS1, perturbing cell polarity thus interfering with epithelial function^6^. Removal of the PBM from SARS-CoV-1 E protein resulted in reduced expression of inflammatory cytokines and pathogenicity in mice^7^. The E proteins of SARS-CoV-1 and CoV-2 differ only in three amino acids and a single residue deletion. Notably, their C-terminal regions demonstrate significant structural plasticity, adopting α-helical or β-sheet conformations depending on the lipid environment, protein concentration, and possibly the bilayer curvature^8,9^. The transition from membrane-bound α-helices to water-exposed β-sheets likely requires a disordered state of the N-terminus of SARS-CoV-2 E protein, and this is also featured in the DisProt database of disordered proteins^10,11^ (https://disprot.org/DP03450).

The SARS-CoV E protein are predominantly confined to the endoplasmic reticulum (ER) and the ER-Golgi intermediate compartment (ERGIC)^12^. The SARS-CoV E proteins can form homopentamers acting as a viroporin that is permeable for ions including calcium^2,13^.

We propose that the E protein acts as an exoregulin of SERCA based on several observations. Firstly, the E protein forms pentamers similar to phospholamban (PLN), a known SERCA regulin. Secondly, these E pentamers function as viroporins, conducting positive ions, including calcium. Additionally, both PLN and the E protein have a single transmembrane (TM) region, a short luminal segment, and a longer intracellular disordered region. Therefore, the structure of the E protein in its monomeric and pentameric forms closely resembles human regulins, which regulate the activity of the sarco/endoplasmic reticulum Ca^2+^ ATPases (SERCA)^14,15^. The major function of SERCA enzymes is to restore the cytosolic Ca^2+^ concentration upon calcium signaling by sequestering Ca^2+^ ions into the ER or sarcoplasmic reticulum (SR). SERCA proteins are encoded by three genes (ATP2A1, 2 and 3) that give rise to several isoforms, among which SERCA2b has the widest tissue expression pattern. This ubiquitous enzyme plays an essential housekeeping role in cellular calcium homeostasis, including that of lung parenchyma and vasculature. Therefore, SERCA has crucial functions in many cellular processes, such as proliferation, contractility, and apoptosis. The role of SERCA-dependent calcium transport in viral infections is receiving increasing attention^16–18^.

SERCA enzymes are modulated by binding of regulins such as phospholamban (PLN) or sarcolipin (SLN) (Fig. 1b)^19^. Some of these interactions reduce the Ca^2+^ affinity of SERCA pumps and the rate of Ca^2+^ uptake into internal Ca^2+^ stores. The two recently discovered regulins, endoregulin (ELN) and the another-regulin (ALN), inhibit the activity of SERCA as PLN^20,21^. ALN exhibits a ubiquitous tissue expression pattern similar to SERCA2b^21^, while ELN is expressed specifically in non-muscle tissues (epithelial cells of trachea and bronchi, lung and intestine)^21^. Their SERCA inhibitory mechanisms are predicted to be similar to PLN due to their similar topology.

The PLN is a 52 amino acid (a.a.) long single-pass membrane protein containing three distinct parts (Fig. 1a). Its N-terminal cytoplasmic region displays structural plasticity. This region is intrinsically disordered in aqueous environments but adopts an α-helical structure when associated with membranes. This characteristic qualifies it as a Membrane Molecular Recognition Feature (MemMoRF)^22^. The structure is further composed of a TM helix, followed by a short C-terminal tail, situated in the ER lumen. PLN was demonstrated to be in dynamic equilibrium between monomeric and pentameric states^23^. The monomer is considered the “active” SERCA-inhibitory form while the pentameric form constitutes the “inactive” PLN pool (Fig. 1c). Oligomer formation was also observed for the other regulins, which were also bound to SERCA in a monomeric form^15^. In addition, different regulins hetero-oligomerize with each other and this may lead to further fine-tuning of their effects on SERCA-dependent calcium transport^24^.

In this study, we investigated whether E protein can act as an exoregulin and interfere with the SERCA system, either by binding directly to SERCA2b or by interacting with endogenous regulins to modulate their monomer/multimer ratio. We also assessed if the interaction between SARS-CoV-2 E protein and SERCA2b has a functional relevance for Ca^2+^ signaling.

## Results

### E protein co-localizes with SERCA2b and its regulins in the endoplasmic reticulum

**Fig. 2:**
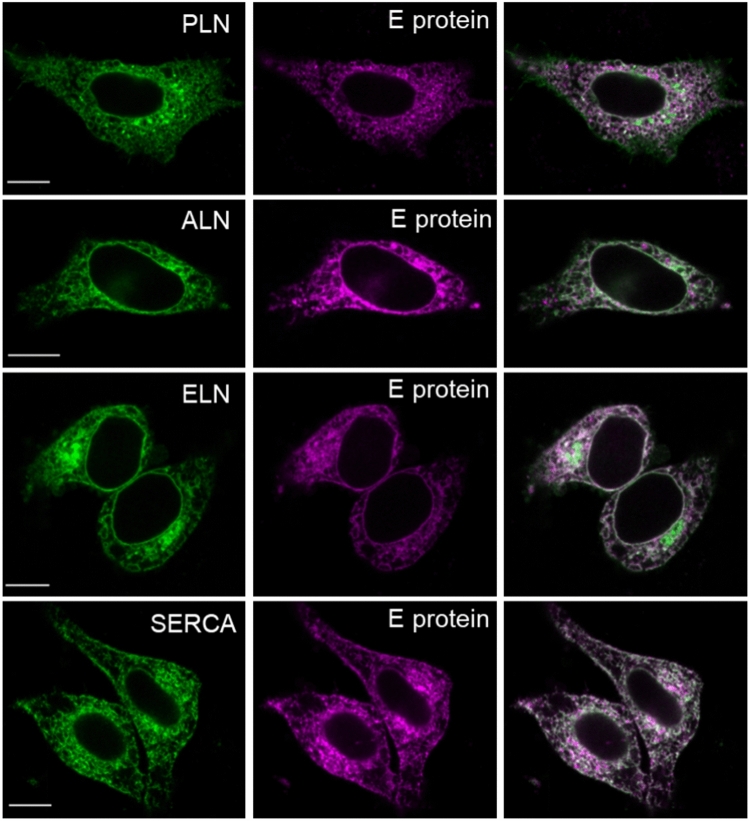
E protein colocalizes with SERCA2b and regulins in the endoplasmic reticulum. Columns with green, magenta, and white indicate the signals from localization of GPF and mCherry constructs and their colocalization, respectively. Scale bar is 10 μm.

To investigate the effect of E protein on the function of the SERCA-system, we co-expressed E protein with SERCA2b or regulins in HeLa cells. The well-known regulator, phospholamban and two non-muscle regulins, ALN and ELN were selected for our studies and were expressed as N-terminally labelled chimeras with eGFP or mCherry. The E protein, expressed as a N-terminal fusion with eGFP or mCherry was localized mainly in the ER and to a lesser extent in the ERGIC compartment after 24 h transfection, based on their co-localization with the immunostained calnexin and ERGIC-53 organellar markers, respectively (Fig. S1). SERCA2b was present in the ER that corresponds to its anticipated location (Fig. 2). Although all regulins were detected in both the ER and the Golgi, their distribution between these two compartments was different. ALN exhibited distinct Golgi localization while PLN and ELN remained mainly in the ER even after 48 h of transfection. E protein labeling indicated that it is predominantly co-localized with PLN, ALN, and ELN regulins, as well as with SERCA2b in the ER (Fig. 2).

### E protein forms heteromers with regulins and SERCA2b

**Fig. 3:**
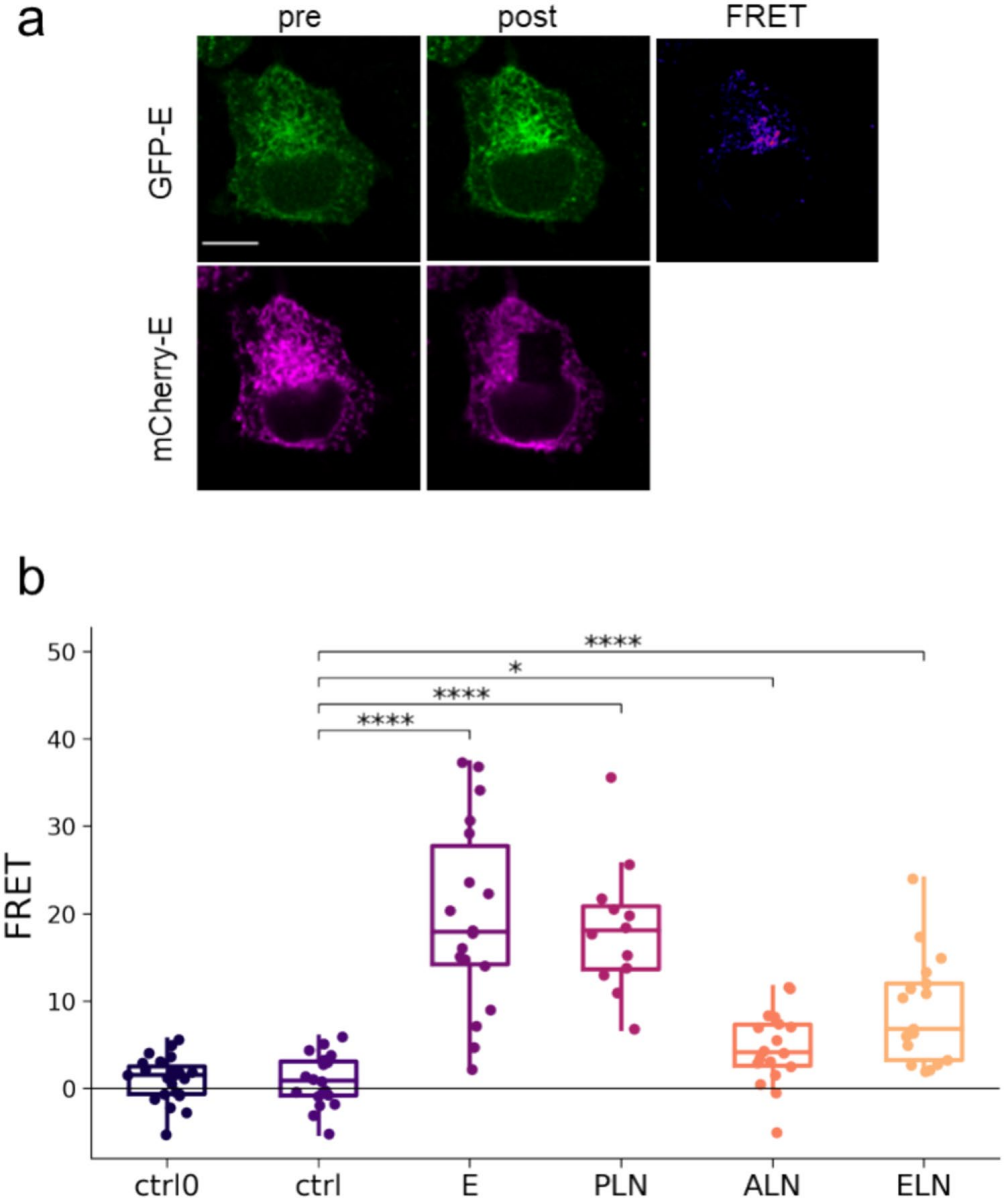
Homo-oligomerizations of E protein and regulins. (**a**) Fluorescence images of HeLa cells expressing eGFP- (green) and mCherry-tagged (magenta) E protein. The first and second columns show images before (pre) and after (post) photobleaching, respectively. The FRET image shows FRET values calculated by *FRETcalc*. The scale bar is 10 μm. (**b**) FRET efficiency values produced by homo-oligomers. ctrl0: eGFP/mCherry pair, ctrl: eGFP-SERCA/mCherry-SERCA pair. Pooled data from 3 to 3 independent experiments are shown. Dots represent individual cells. Median and interquartile ranges are indicated with box plots. Data were analyzed by Kolmogorov–Smirnov test (*p < 0.05, ****p < 0.0001).

Since co-localization does not necessarily indicate interaction, first we used Förster Resonance Energy Transfer (FRET) to study further the association between the E protein and members of the SERCA/regulin system. FRET was monitored by acceptor photobleaching (AP-FRET) technique using donor eGFP and acceptor mCherry (Fig. 3a). Homo-oligomerization of E protein and regulins is a well-known phenomenon, and we confirmed homo-oligomer formation of both E protein and regulins in our system (Fig. 3b). The highest and lowest FRET values were observed for the PLN-PLN and ALN-ALN multimers, respectively. The lower ALN values, which still can be considered significant when compared to the control measurements with eGFP-SERCA/mCherry-SERCA pairs, are probably due to the interfering effect of unlabeled endogenous ALN of HeLa cells (https://www.proteinatlas.org/ENSG00000164096-C4orf3/cell+line). This labeled SERCA pair was used as negative control, since the SERCA2b isoform is not expected to oligomerize at lower abundance, which was confirmed by similar negative FRET values of a second control, the soluble eGFP/mCherry pair (Fig. 3b, ctrl0).

**Fig. 4:**
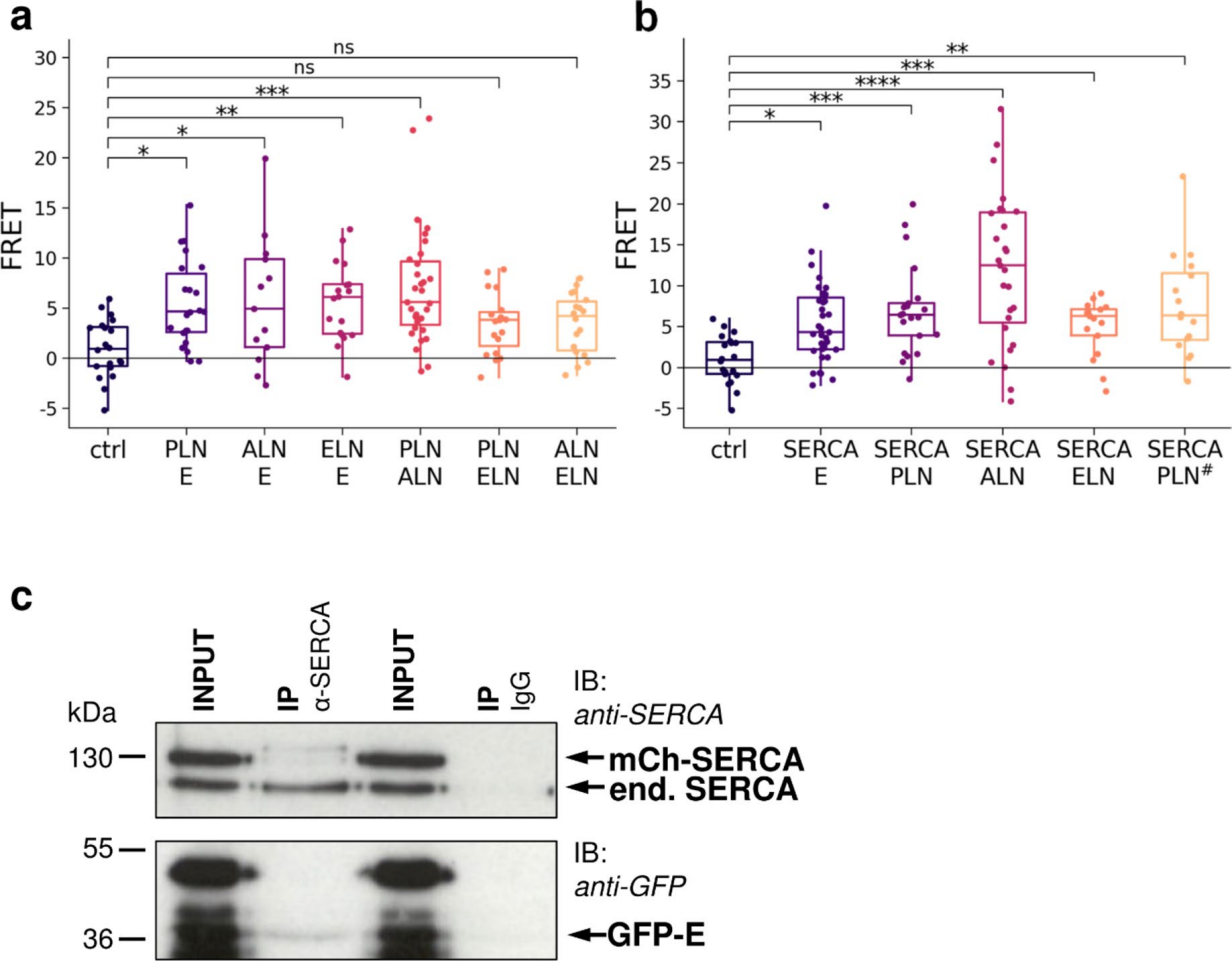
FRET efficiency for hetero-oligomerization among E protein, regulins (**a**), and SERCA (**b**). Ctrl: eGFP-SERCA/mCherry-SERCA pair. PLN^#^: FRET values for eGFP-PLN tagged at the luminal end and mCherry-SERCA as a positive control for transmembrane measurements). Pooled data from 3 to 3 independent experiments (for SERCA/E pair: 5 independent experiments) are shown. Dots represent individual cells. Median and interquartile ranges are indicated with box plots. Data were analyzed by Kolmogorov–Smirnov test (*p < 0.05, **p < 0.01, ***p < 0.001, ****p < 0.0001, ns: not significant). (**c**) The immunoprecipitation experiment using the anti-SERCA IID8 antibody successfully confirmed an association between the eGFP-E protein and mCherry-SERCA2b. Western blot analysis was employed for both the lysates (INPUT) and the eluted fractions (IP), using anti-SERCA IID8 antibody and anti-GFP antibodies as indicated. Non-immune mouse IgG1 was used for IP as a negative control (lanes 3 and 4). Input is ~ 1% of the sample loaded for the immunoprecipitation (~ 1 µg).

Since heteromerization of PLN and SLN has been described and has been also suggested for other regulins^15^, we investigated the hetero-oligomerization for PLN–ALN, PLN–ELN and ALN–ELN pairs in our system. Although heteromerization of these constructs was indicated by significantly increased FRET levels (Fig. 4a), the values were dispersed. No or low FRET was observed in some cells and a markedly high FRET signal was detected in other cells. This phenomenon was likely the result of the possible hetero-oligomerization combinations of two labeled proteins (“Supplementary Text”). We also observed increased FRET signals between E protein and regulins (Fig. 4a). The significance of these high values for E protein heteromers are underscored by the fact that only the E protein tagged at its N-terminal (luminal) end resulted in a stable fluorescent protein, thus eGFP and mCherry gave FRET signal despite that they localized at different sides of the ER membrane. It is worth mentioning that the thickness of the membrane bilayer (30–50 Å) is known to allow FRET signal detection between eGFP and mCherry (Förster radius ~ 51 Å) (“Supplementary Text”). To confirm being able to detect transmembrane FRET, we generated a PLN protein fluorescently labelled at the luminal end and measured FRET with SERCA labelled at the cytosolic side, as they are known to form a complex and used them as positive control (Fig. 4b, SERCA/PLN# pair). We observed similar FRET values both for PLN labeled at the cytosolic side or labeled at the luminal end. This result can be explained by the structure of PLN and its localization in the PLN/SERCA complex. Compared to the cytosolic region of PLN, the region in the ER lumen is very short and, as a result, the two ends of the PLN in the SERCA/PLN complex are at similar distances from the N-terminus of SERCA where other fluorescent tag is located.

We also studied the E protein interaction with SERCA2b and found that the FRET values were significantly higher than those of the eGFP-SERCA and mCherry-SERCA control pair (Fig. 4b). These FRET values of E-protein/SERCA were similar to that measured for regulins/SERCA interactions. The exception was the SERCA and ALN pair, which exhibited the highest FRET (Fig. 4b). Importantly, the fluorescent proteins were also located on the opposite sides of the membrane bilayer in the case of the E protein/SERCA complex indicating strong interaction between these proteins.

To further confirm an interaction between SERCA and the E protein beyond the spatial proximity indicated by FRET, we employed co-immunoprecipitation (Fig. 4c). We transfected HeLa cells with eGFP-tagged E protein and mCherry-SERCA and subsequently immunoprecipitated SERCA using the anti-SERCA2 IID8 antibody. The Western blot analysis of the immunoprecipitates, using an anti-eGFP antibody, revealed a distinct 36 kDa band corresponding to the eGFP-tagged E protein. Multiple bands observed in the input cell lysate can be attributed to two factors: The E protein typically exhibits multiple bands due to post-translational modifications and oligomerization^25^, and some of the GFP tags were subject to cleavage. Notably, the 36 kDa band, representing the full-length eGFP-E protein construct, was predominantly immunoprecipitated, while the other species were not. Since our results showed that HeLa cells express high levels of endogenous SERCA compared to exogenous mCherry-SERCA, we repeated the co-immunoprecipitation experiment with cells transfected with eGFP-E protein alone. Using the anti-SERCA IID8 antibody, we were able to pull down GFP-E protein through the interaction between endogenous SERCA and eGFP-E protein (Fig. S2). The co-immunoprecipitation of E protein with SERCA2 thus provides further evidence for an interaction or association between SERCA2 and the E protein.

### Structure and dynamics of E protein complexes

**Fig. 5:**
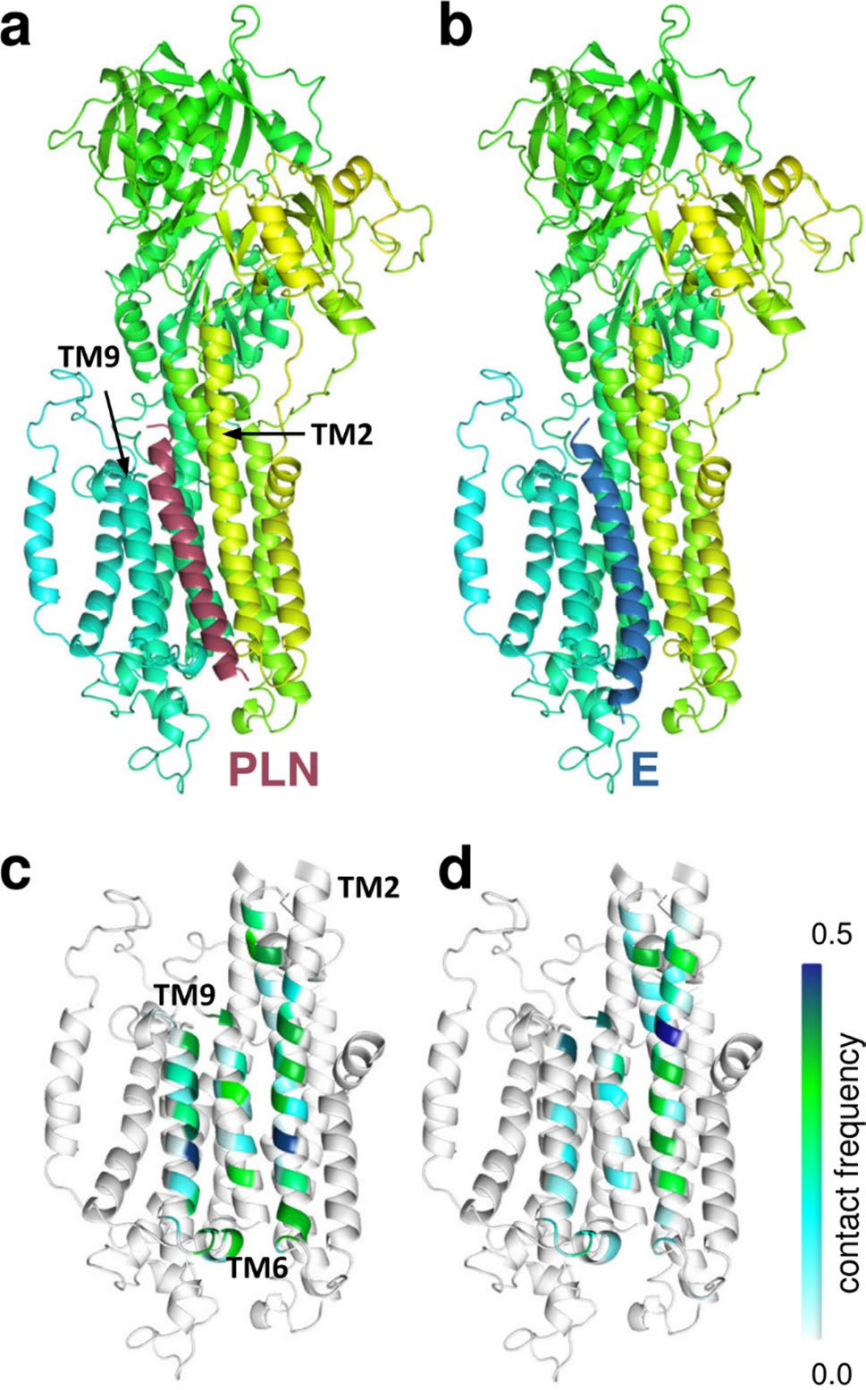
E protein binds to SERCA similarly to PLN. (**a**) PLN (burgundy) in the model of the human SERCA/PLN complex and (**b**) E protein (blue) from PIPER/ClusPro docking. SERCA is colored yellow-green-turquoise. The interaction frequency (white to blue) of (**c**) PLN and (**d**) E protein calculated from simulations are projected to SERCA TM helices.

We aimed to characterize the structure and dynamics of complexes using the tools of 3D-bioinformatics and computational biology. Although we were able to use AlphaFold-Multimer to predict the complexes of the plasma membrane Ca^2+^ ATPase (PMCA), which is a close relative of SERCA and its obligatory 1 TM partner proteins (PMCA/basigin and PMCA/neuroplastin)^26^, AlphaFold-Multimer was not able to build a rational structure for complexes including SERCA and regulins (Fig. S3). This could be attributed to the transient nature of SERCA interactions, and a consequently low evolutionary information encoded on the protein–protein interaction interface, insufficient for successful AlphaFold predictions. Therefore, we used PIPER/ClusPro^27^ to generate the structures of these complexes, and this resulted in docked E protein and regulins being located at the same site as observed in the SERCA/PLN experimental structure (Fig. 5).

In order to compare the stability of the E protein and PLN interactions with SERCA at a higher detail, we performed molecular dynamics (MD) simulations with these complexes (3 × 500 ns for each complex) embedded in a lipid bilayer (Fig. S4). The PLN and E protein exhibited overlapping SERCA binding sites, interacting with SERCA TM2, TM6, and TM9 (Fig. 5c,d). However, E protein interactions were less frequent with most of the TM6 and TM9 residues and its most stable contacts were formed with residues at the intracellular bilayer boundary of these helices. The novel AlphaFold3 generated a reasonable structure of the SERCA/E protein complex, in which SERCA exhibits a different conformation and E protein interacts with TM2 (Fig. S5).

We also aimed to model the homo- and hetero-oligomers of regulins and E protein, and characterize their dynamics with MD simulations. However, we found that the E protein pentamer with a pore was unstable in simulations (Fig. S6), exhibiting movements previously reported for regulins PLN and SLN^27^. AlphaFold-Multimer generated a model for the E protein pentamer, albeit with low confidence, contrasting with the high-confidence monomer model (Fig. S2). This monomer model, particularly in the TM region, demonstrated high pLDDT scores, aligning well with experimental data. Notably, lower pLDDT scores were observed for the two C-terminal, membrane-interacting helices, which exhibited unfolding in MD simulations^28^.

### E protein causes unstable ER Ca^2+^ dynamics

**Fig. 6:**
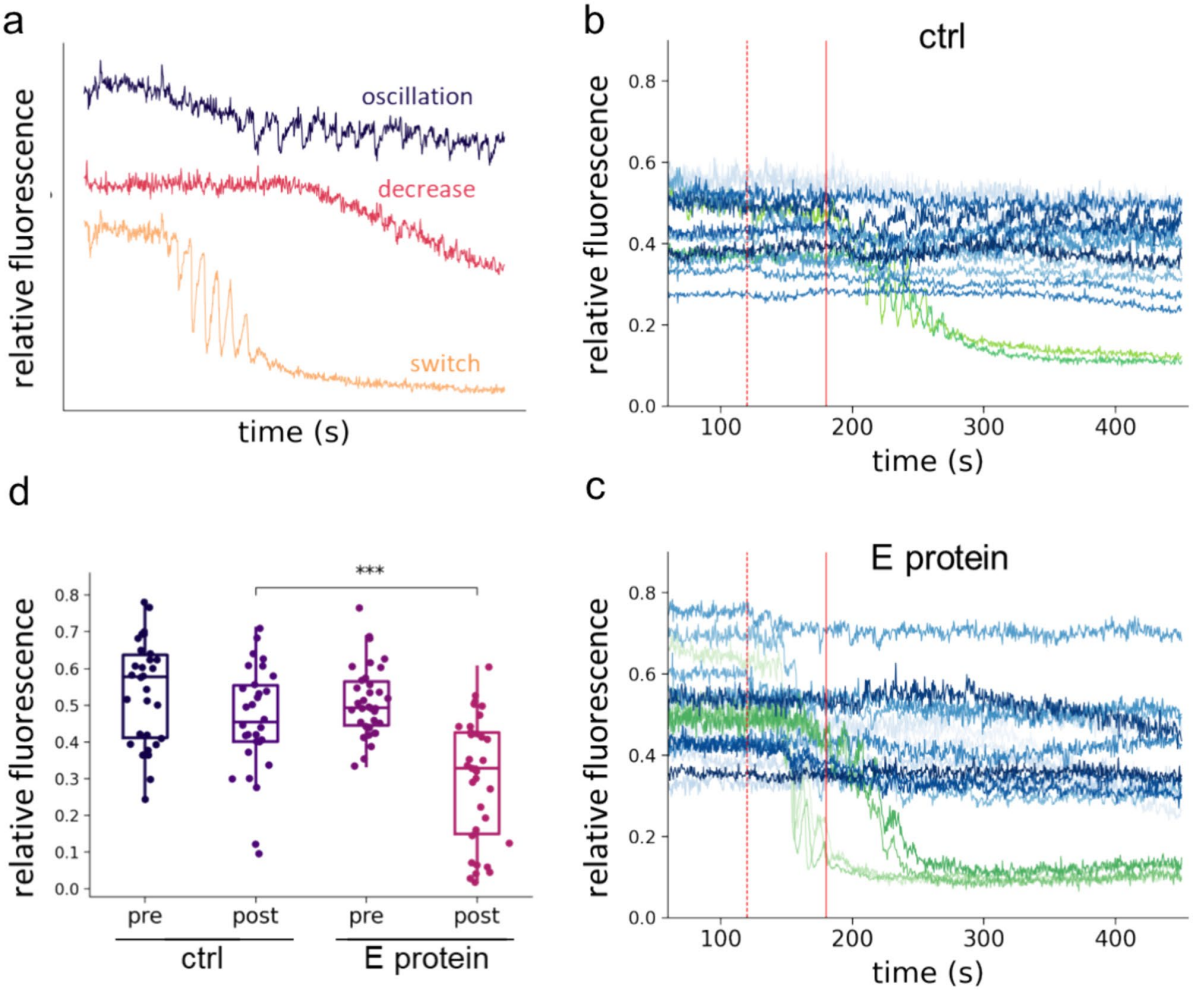
ER Ca^2+^ dynamics triggered by ATP. (**a**) Response types in the ER Ca^2+^ signal after addition of ATP. (**b**,**c**) Representative Ca^2+^ level changes in cells expressing ER-GCaMP6-150 sensor alone (**b**) and with E protein (**c**) caused by ATP. Curves show the signals of individual cells. To better display the switch-like responses, this type of response is highlighted in green as opposed to other responses, which are blue. Red dashed (200 µM) and solid (400 µM) lines indicate ATP addition. (**d**) Ca^2+^ level before ATP treatment (pre) and at 5 min after ATP addition (post) (ctrl: n = 30, E protein: n = 30, from 3 to 3 independent experiments). Data were analyzed by Wilcoxon–Mann–Whitney test (***p < 0.001).

To explore the functional significance of the interaction between E protein and SERCA2b, we investigated whether this interaction had an effect on the ER Ca^2+^ homeostasis first. The ER Ca^2+^ level was monitored by using the genetically encoded ER-specific calcium indicator, the ER-GCaMP6-150^28^. To monitor SERCA2b activation the purinergic receptors were stimulated by extracellular ATP. First, ATP was administered to generate intracellular IP_3_ production leading to the activation of the IP_3_-sensitive ER Ca^2+^ channels and Ca^2+^ release from the ER lumen. ATP was applied at two concentrations (240 μM and 400 μM) at 2 and 3 min of the experiments in the presence of physiological [Ca^2+^], and the change in ER [Ca^2+^] was monitored in the absence and in the presence of mCherry-tagged E protein. Three different responses were observed in individual HeLa cells (Fig. 6a). We observed a switch-like response in several cells that caused a rapid and significant decrease in ER calcium levels, which was frequently accompanied by Ca^2+^ oscillation. The second and third types of cellular response were the initiation of Ca^2+^ oscillation and a slow decrease in ER Ca^2+^ levels, respectively (Fig. 6a). In control cells, the switch-like response occurred only in response to high ATP concentrations and was not as prompt as in cells expressing E protein (Fig. 6b,c). Simple decrease was the dominant response (65%). 28% of cells showed an oscillatory response and only 7% showed a switch-like response. In contrast, in cells expressing E protein the ratio of switch-like responses was increased (32%) while the number of oscillatory responses decreased (9%). The ratio of the “slow decrease” answer did not change substantially (59%).We evaluated the change in ER Ca^2+^ levels by comparing the ER Ca^2+^ levels before and 5 min after ATP administration. Following ATP administration, significantly lower ER Ca^2+^ levels were detected in the presence of E protein than in its absence (Fig. 6d). In addition, strong switch-like responses corresponding to a sudden depletion of the ER store were observed more frequently in cells expressing E protein than in control cells. The rapid response to ATP and lower ER Ca^2+^ levels in the presence of E protein suggest that this viral protein sensitized the ER Ca^2+^ homeostasis for calcium depletion. Whether changes in passive Ca^2+^ permeability of ER membrane and/or suppression of the SERCA activity accounts for the observed phenomenon, was addressed next.

### E protein over-expression does not alter the ER Ca^2+^ passive permeability

**Fig. 7:**
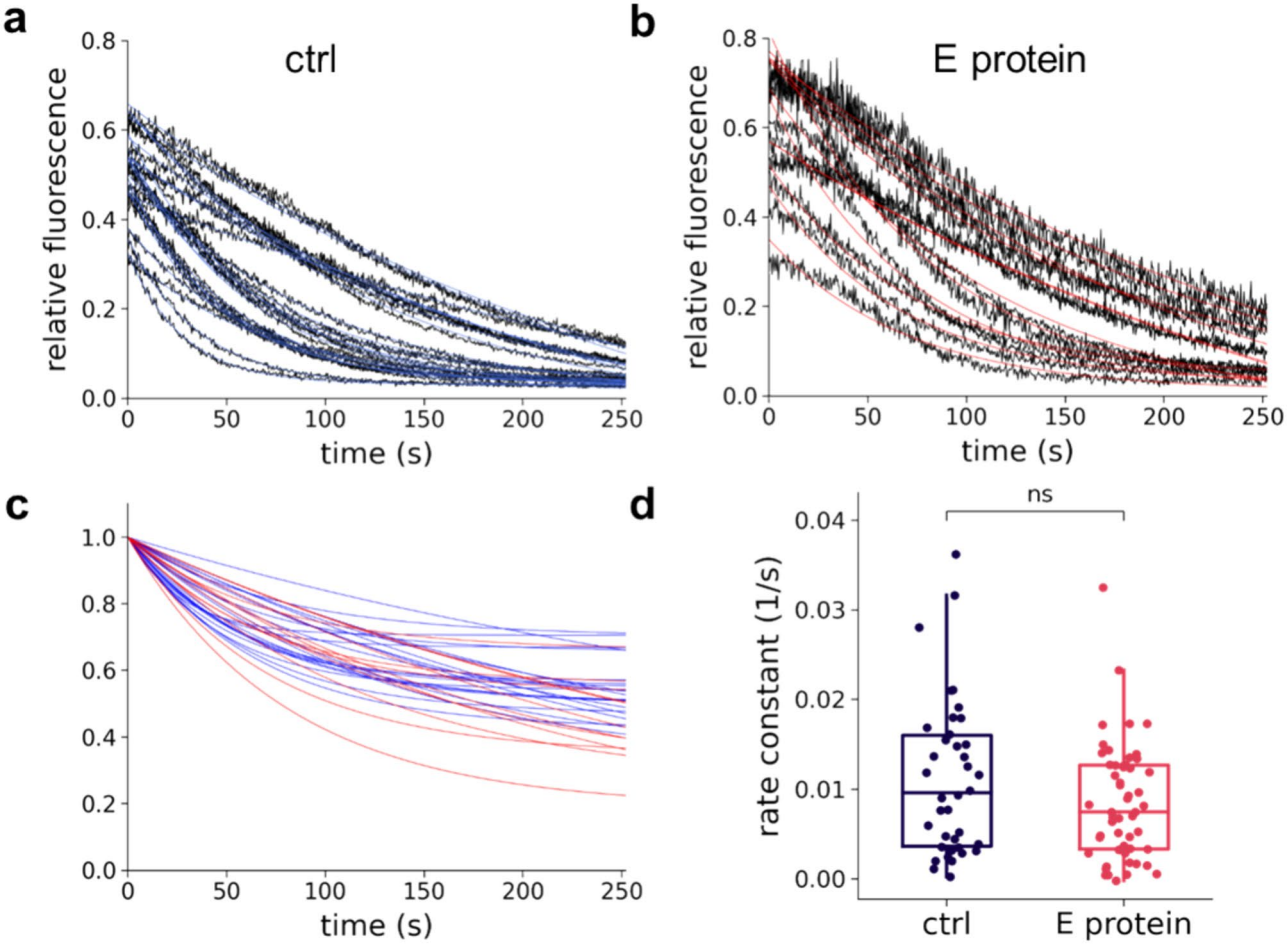
E protein does not alter Ca^2+^ leakage from the ER. Representative traces of Ca^2+^ leakage from the ER after SERCA inhibition by thapsigargin, monitored with the ER-GCaMP6-150 Ca^2+^ sensor. The graphs show the change in [Ca^2+^]_ER_ after Tg addition in the absence (control) (**a**) and in the presence of E protein (**b**). Single exponential decay functions were fitted to the data points (blue and red curves) to determine the leakage rate constant. (**c**) For comparison of leakage kinetics, the starting points of the fitted curves are shifted to coordinates (0, 1). Red indicates the leakage in the presence of E protein, blue indicates the control measurements without E protein. (**d**) Box plot of Ca^2+^ leakage rate constants in control (n = 38) and E protein (n = 54) expressing cells (from 3 to 3 independent experiments; ns: not significant; Kolmogorov–Smirnov test).

E protein is known to form homo-pentamers, providing a cation-selective pore^29^. Since MD simulations suggested that the pore of the pentamer is not stable (Fig. S4), we investigated whether E protein was able to affect Ca^2+^ balance between the ER and the cytoplasm through mechanisms independent of its viroporin function. Therefore, we inhibited SERCA-mediated ER refill by thapsigargin (Tg) and monitored Ca^2+^ efflux from the ER, in the presence and absence of mCherry tagged E protein, detected by the decrease in ER-GCaMP-150 fluorescence. The continuously decreasing ER Ca^2+^ levels measured in individual cells were fitted with exponential curves to obtain the leakage rate constant (Fig. 7). Considering that the rate constants did not exhibit significant differences in the absence and presence of E protein, our results indicated that E protein did not affect the passive Ca^2+^ leakage from the ER suggesting that it did not conduct Ca^2+^ in our cellular system.

### E protein over-expression alters the reload into the ER

**Fig. 8:**
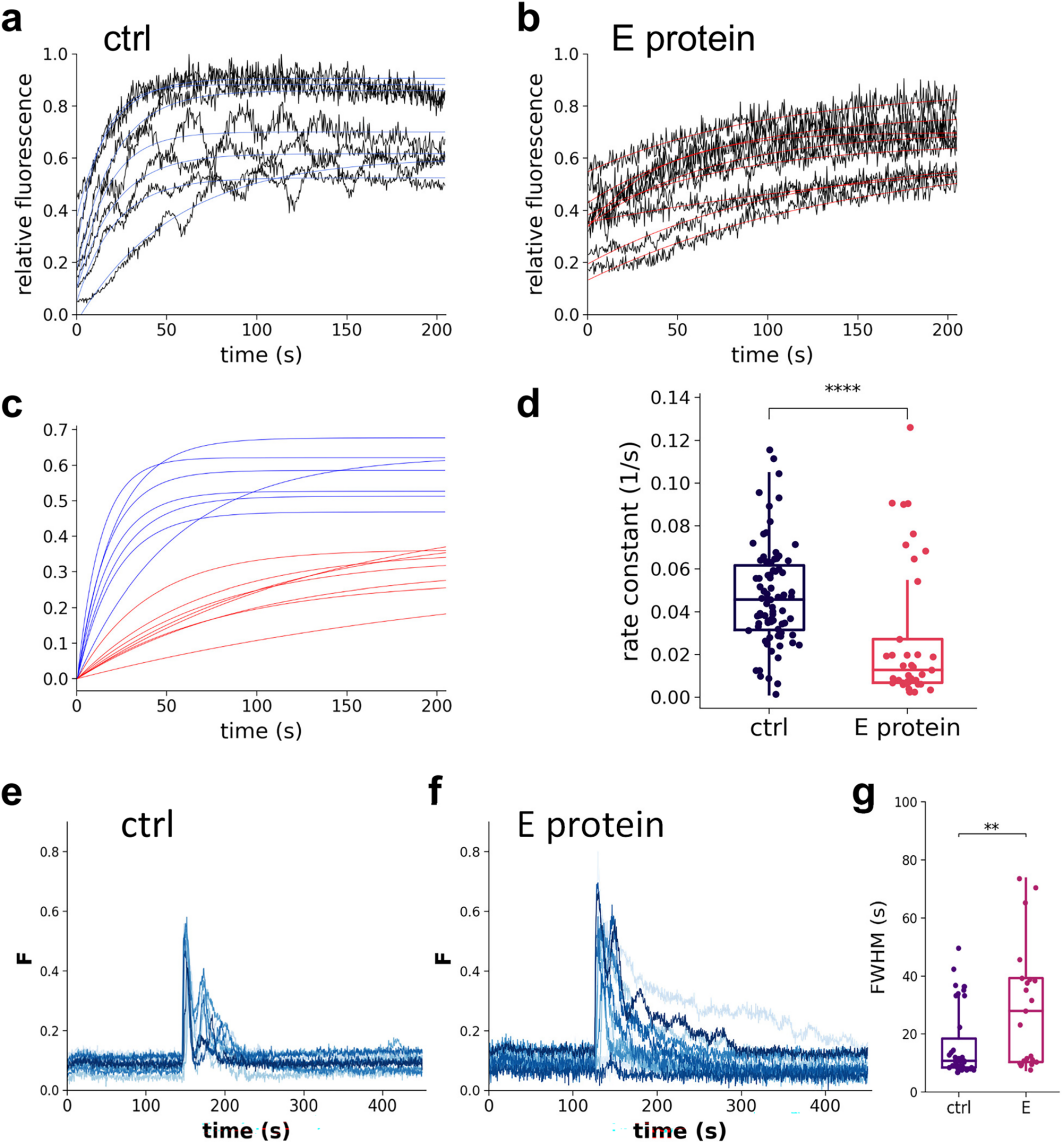
E protein alters the Ca^2+^ reload into the ER. Representative traces of Ca^2+^ reload into the ER after store depletion in the absence (control; ctrl) (**a**) and in the presence of E protein (**b**). Single exponential growth functions were fitted to the data points (blue and red curves) to determine the reload rate constant. (**c**) For comparison of reload kinetics, the starting points of the fitted curves are shifted to coordinates 0.0. (**d**) Box plot of Ca^2+^ reload rate constants from control (n = 83) or E protein (n = 37) expressing individual cells. Median and interquartile range are indicated (from 4 to 4 independent experiments). Data were analyzed by Kolmogorov–Smirnov test (****p < 0.0001). (**e**,**f**) Representative cytosolic Ca^2+^ level changes upon ATP addition in cells stably expressing GCaMP2 sensor alone (**e**) and co-expressing with E protein (**f**). Curves show the relative fluorescence of cytosolic Ca^2+^ sensor in individual cells. (**g**) Full width at half maximum (FWHM) of Ca^2+^ peaks in control and E protein expressing cells (ctrl: n = 39, E protein: n = 25, from 3 to 3 independent experiments). Data were analyzed by Wilcoxon–Mann–Whitney test (**p < 0.01).

To assess whether the E protein overexpression can interfere with the ER Ca^2+^ uptake, we monitored the rate of ER Ca^2+^ accumulation following the depletion of the ER Ca^2+^ store. The ER Ca^2+^ was depleted by activation of the IP_3_ receptor following ATP-induced IP_3_ generation in Ca^2+^-free medium. Then, the extracellular medium was replaced with a medium containing 2 mM Ca^2+^ to initiate Ca^2+^ uptake from the cytosol, catalyzed by SERCA^30^. The increase in the ER Ca^2+^ concentration was significantly slower and the rate constant of this increase was significantly lower in cells expressing E protein than in the control cells (Fig. 8a–d) strongly suggesting that E protein affects SERCA function. To check whether Ca^2+^ reuptake was measured under identical initial Ca^2+^ gradients between the cytosol and ER compartment in the absence or presence of E protein, we compared resting fluorescence values of the ER Ca^2+^ sensor and the cytosolic Ca^2+^ sensor (Fig. S7). There were no significant differences between these values, indicating that the resting gradient remained unchanged upon E protein expression. Since E protein did not increase Ca^2+^ leakage in our system (Fig. 7) and SERCA plays an exclusive role in ER Ca^2+^ uptake, confirmed by the complete stop of ER reloading by using SERCA inhibitor thapsigargin (Fig. S8), our results strongly suggest that E protein inhibits the function of SERCA.

### Cytosolic Ca^2+^ dynamics is altered by E protein expression

Although the study of the ER Ca^2+^ signal gives the most direct information about SERCA activity, we also recorded changes in the cytosolic Ca^2+^ concentration, a critical determinant of cellular responses. Control cells and cells transfected with E protein were stimulated with ATP as in experiments targeting the ER Ca^2+^ dynamics. However, this time we monitored the cytosolic Ca^2+^ signal using a HeLa cell line stably expressing the GCaMP2 cytosolic Ca^2+^ sensor^31^. We found that a lower percentage of cells responded to ATP with developing the usual cytosolic calcium signal when E protein was expressed, compared to control cells. 91% of control cells exhibited a Ca^2+^ peak, while response to ATP was observed only in 66% of E protein expressing cells.

The Ca^2+^ signal decay in the presence of E protein were slower (Fig. 8e–f). We used the full width at half maximum (FWHM) values to characterize these signals, which confirmed slower signal decay in the presence of E protein (Fig. 8g). Given that the rate of decline in cytosolic [Ca^2+^] during transients is largely dependent on SERCA activity, these findings are consistent with an inhibitory effect of the E protein on SERCA function.

## Discussion

Our results demonstrate that SARS-CoV-2 E protein interacts with members of the SERCA regulatory system, including SERCA2b as well as regulins (Fig. 1c) based on FRET and co-immunoprecipitation experiments (Figs. 3, 4). The relevance of SERCA/E protein interaction was confirmed by studying the Ca^2+^ dynamics of the ER. We found that in the presence of E protein, the ATP-induced ER Ca^2+^ signal was perturbed and this dysfunction also affected the cytosolic Ca^2+^ signal. This phenomenon was not due to a viroporin-mediated Ca^2+^ channel function of the E protein, but rather an inhibition of SERCA by the E protein, reducing ER Ca^2+^ refill. Similar functional consequences of E protein on Ca^2+^ signaling in Fig. 2 in^32^ support our findings, although, this was not explored further in that work.

By using AP-FRET we confirmed spatial proximity of E protein, regulins and SERCA2b. Moreover, our results on regulin heterodimerization are in good agreement with a recent study on regulins with FRET-based experiments^15,24^. Most importantly, our FRET and immunoprecipitation experiments demonstrated a tight interaction or association between E protein and SERCA, confirming high-throughput assays, which also identified this interaction^33,34^.

Interestingly, the highest FRET among SERCA–regulin pairs was measured for SERCA2b-ALN. Since the tissue- and cell-type specific expressions of this pair exhibit a remarkable overlap, these proteins can be considered as a semi-obligate pair^21^. To our knowledge, we investigated the interactions of the SERCA2b isoform with regulins using FRET for the first time. It should be noted that the molecular-level structural matching of the different SERCA isoforms and regulins inferred from the FRET results is crucial, especially in systems such as the heart, where multiple SERCA isoforms (SERCA2a, SERCA2b) and multiple regulins (PLN, sarcolipin, ALN) are coexpressed^15^.

E protein is known to form a pentameric structure providing an ion channel^2^. Regulins are also known to form pentamers in the ER membrane creating a pool of oligomers, which is in dynamic equilibrium with the monomeric pool, directly involved in SERCA regulation. Therefore, our results on E protein/regulin heterodimerization strongly suggest that E protein can interfere with SERCA-regulation at multiple levels. Although the MD simulations suggested a collapse of the E protein pentamer (Fig. S4), and no ER Ca^2+^ efflux through the E protein was observed in our experiments (Fig. 7), we believe that the expression levels of the transfected E protein in our system are sufficient for potential oligomeric channel formation. However, it is possible that the fluorescent tags attached to the transfected E protein might interfere with this process. Nonetheless, the delayed formation of the E protein viroporin, as observed in other studies, could be a crucial mechanism allowing the virus to avoid premature cell death and ensure successful viral replication.

Importantly, the virally reprogrammed Ca^2+^ signaling can lead to different cell fates depending on the type of the virus or the stage of viral replication. For example, Ca^2+^ depletion may delay apoptosis to gain time for replication (enteroviruses)^35^ or it may promote apoptosis, facilitating virion release (hepatitis C)^36^. Recently, the role of SERCA in viral infections has become an intensively studied topic, related to the regulation of autophagy and inflammatory responses^37^. Both of these processes are influenced by SERCA regulation via Vacuole Membrane Protein 1 (VMP1)^37^, which plays a key role in viral (SARS-CoV-2) infections^2,37^. The VMP1, similarly to E protein, can interact with both SERCA and regulins (PLN and SLN), but these interactions activate SERCA, since VMP1 counteracts SERCA/regulin inhibitory interactions^38^. E protein may also interfere with SERCA regulation at this SERCA/VMP1 level, supported by observations of interactions between VMP1 and the E protein in high-throughput studies^39^.

In summary, our results demonstrate that the SARS-CoV-2 E protein modulates host cell Ca^2+^ homeostasis by affecting the SERCA–regulin system. The inhibitory effect was not exerted by Ca^2+^ conductance through E protein, but through a direct interaction between E protein and SERCA2b, possibly involving a tripartite interaction including competing regulins. It is conceivable that important pathophysiological steps caused by viral infection, including autophagy and inflammatory responses, which are hallmarks of severe COVID-19 cases, may be rescued through the manipulation of SERCA activity. Therefore, it is of crucial importance to explore the infection-associated chain of calcium signaling events in detail for enabling therapy.

## Methods

### Constructs and chemicals

For mammalian expression of eGFP-labelled proteins we used the pEGFP-C1 (Clontech) vector. For mCherry labelling we replaced eGFP to mCherry from pTK96_mCherry-MRLC2 (Addgene #46358) vector using AgeI and BsrGI sites. To construct the pEGFP-C1_SERCA vector carrying an N-terminally eGFP-labelled human SERCA2b, we amplified the SERCA2b cDNA from Addgene plasmid #75188 using SERCA specific forward 5′-GGGAGATCTATGGAGAACGCGCACACCAAGACGG-3′ and reverse 5′-GGGGTCGACTCAAGACCAGAACATATCGCTAAAGTTAG-3′ primers with overhanging BglII and SalI sites for pEGFP-C1 vector insertion. cDNAs of human regulins and SARS-CoV-2 E protein with flanking BglII and BamHI restriction sites were custom synthesized (Integrated DNA Technologies; pIDTSMART-KAN vector; ALN: NM_001001701.3; ELN: NM_001162997.1; PLN: NM_002667.4, E protein: NC_045512.2). Vectors expressing N-terminally fluorescently labeled regulins and E protein were created by moving the cDNA of regulins into pEGFP-C1 and pmCherry-C vectors with BglII and BamHI.

ER-GCaMP6-150 cDNA was amplified from the Addgene plasmid #86918 using the ERGcAMPEcofw (5′-GGGGAATTCTCACAGCTCATCCTTG-3′) and ERGcAMPNotrev (5′-TTTGCGGCCGCATGGGACTGCTGTCT-3′) primers, digested with EcoRI and NotI, and inserted into the pSB-CMV-CAGPuro vector (gift of Tamás Orbán). All constructs were verified by Sanger sequencing.

### Cell culture and transfection

HeLa cells (ECACC, 93021013) were grown in Dulbecco’s modified Eagle’s medium (DMEM) supplemented with 10% Fetal Bovine Serum (FBS) and Penicillin/Streptomycin at 37℃ in 5% CO_2_. The cells were seeded into an eight-well Nunc Lab-Tek II chambered cover glass (No. 155409) at 4 × 10^4^/well density one day prior to transfection. Cells were transfected with the appropriate DNA constructs using FuGENE HD transfection reagent according to the manufacturer’s protocol. For co-transfection experiments, the plasmids were used at a ratio of 1:1. For FRET experiments, cells were fixed with 4% paraformaldehyde in PBS for 15 min at 37 °C.

### Immunostaining and fluorescent microscopy

For co-localization studies, the endogenous ERGIC-53 protein was stained by the anti-ERGIC-53 antibody (Sigma, E1031; ERGIC marker) and the ER marker, calnexin was stained by anti-calnexin (Sigma, C4731; ER marker). One day after transfection, cells were washed with phosphate-buffered saline (PBS) and fixed with 4% paraformaldehyde in PBS for 15 min at 37 °C. Cells were permeabilized in pre-chilled (− 20 °C) methanol for 5 min. Samples were blocked for 1 h at room temperature in PBS containing 2 mg/ml BSA, 0.1% Triton X-100 and 5% goat serum, then incubated for 1 h at room temperature with primary antibodies diluted in blocking buffer containing 2 mg/ml bovine serum albumin (A7030, Sigma-Aldrich), 0.1% Triton X-100 (X100PC, Sigma-Aldrich) and 5% donkey serum (S2170-100, BioWest). After washing with PBS, cells were incubated for 1 h at room temperature with Cy3-conjugated secondary antibody diluted 250× in blocking buffer. After repeated washes, samples were studied with a Nikon Eclipse Ti2 using 60 × oil immersion objective. Green and red fluorescence was acquired at 505–550 nm and > 580 nm, using excitations at 488 and 561 nm laser lines, respectively.

### Confocal microscopy and acceptor-photobleaching (AP) FRET measurements

Cells were washed with phosphate-buffered saline (PBS) and fixed with 4% paraformaldehyde in PBS for 15 min at 37 °C and washed 3× with PBS. Co-localization studies and AP-FRET was performed with a Nikon Eclipse Ti2 confocal microscope with a 60× oil immersion and/or 20× objective. For AP-FRET, eGFP fluorophore was used as donor, and excited with the 488 nm laser line. The emission was collected between 505 and 550 nm. As acceptor, mCherry fluorophore was used, which was excited with the 561 nm laser line. The emission was collected above 580 nm. A region of interest (ROI) was selected within the ER compartment and 100% intensity of the 561 nm laser for 10 iterations was used for photobleaching the acceptor/mCherry to background levels. Pre-bleach and post-bleach images were acquired using identical imaging settings. FRET efficiency was calculated by the FRETcalc (v5.0) ImageJ (v1.53p) plugin allowing pixel-by-pixel analysis which is especially suitable for the analysis of proteins expressed in the endoplasmic reticulum^40^.

### Calcium signaling measurements

After 24 h of culture in 8-well chambered cover glass, cells were transfected with calcium indicator ER-GCaMP6-150 with or without mCherry-E protein. 24 h after transfection, medium was replaced by Hanks’s Buffered Salt Solution (HBSS, Thermo Fischer 88284) supplemented with 10 mM HEPES (pH 7.4) and 2 mM CaCl_2_.

Three types of experiments were performed. (a) ATP stimulation: cells were stimulated with 240 μM and 400 μM ATP at 2 and 3 min, respectively in the presence of 2 mM extracellular Ca^2+^. (b) ER leakage: The extent of ER efflux was monitored under SERCA inhibition with 5 μM thapsigargin in the presence of 2 mM Ca^2+^. (c) The ER refill was measured using “Ca^2+^ re-addition” protocol with slight modifications^31^. Intracellular Ca^2+^ stores were depleted by 400 μM ATP treatment in Ca^2+^-free HBSS (Capricorn, HBSS-2A) supplemented with 10 mM HEPES (pH 7.4), 100 μM CaCl_2_ and 100 μM EGTA. After depletion, 2 mM Ca^2+^ was added and the progress of ER Ca^2+^ reload was monitored. Fluorescence changes of the ER-GCaMP6-150 sensor were analyzed using the ImageJ Time Series Analyzer (v2.0) plugin. The maximum and minimum values were obtained with 250 μM ionomycin and 2 mM EGTA, respectively, then the signals were normalized between 0 and 1.

### Co-immunoprecipitation (Co-IP)

HeLa cells were transfected for 24 h with mCherry-SERCA and eGFP-E protein constructs. Immunoprecipitation was carried out using Pierce™ Co-Immunoprecipitation Kit (Pierce, CAT# 26,149). Following the kit protocol, cells were lysed on ice using the kit’s lysis buffer. The lysis buffer contained 1% NP-40. The cell lysate was centrifuged at 13,000×*g* for 10 min at 4 °C. SERCA was bound from the resulting lysate using resin beads treated with IID8 antibody (Sigma, S1439) specific for SERCA2. Non-immune mouse IgG1 (Sigma, M5284) was used as a negative, non-specific control. Bound proteins were eluted, and Western blot analysis was performed with anti-SERCA IID8 (Sigma, S1439) and anti-GFP (Aves, GFP-1020) antibodies staining mCherry-SERCA and GFP-E protein, respectively.

### Western blot analysis

Western blotting was used to verify protein expression and co-immunoprecipitation. Proteins were separated by electrophoresis on 10% SDS polyacrylamide gels. Following electrophoresis, proteins were transferred to a polyvinylidene difluoride (PVDF) membrane. All non-specific binding sites were blocked by incubating membranes in blocking solution, 5% non-fat dry milk in Tris-Buffered Saline, Tween (TBST) for one hour. Next, the membranes were incubated in milk-TBST containing the primary antibody for an hour (anti-GFP, Aves, GFP-1020) or overnight (anti-mCherry, Rockland, 600-401-P16). After washing, the membranes were incubated in milk-TBST containing secondary antibody for one hour. Depending on the imaging methods, we used horseradish peroxidase-conjugated (HRP) secondary antibodies (anti-rabbit, Jackson immunoResearch, 111-035-003, anti-mouse, JacksonImmunoResearch, 115-035-003) or fluorescence-tagged secondary antibody (Cy3 goat anti-rabbit IgG, Thermo Fischer Scientific, A10520). After 5× of washing, HRP-antibody-antigen complexes were detected with SuperSignal™ West Pico PLUS Chemiluminescent Substrate (Thermo, Scientific, 34580) using Curix 60 processor (AGFA). In the case of the fluorescence labelled antibodies Typhoon biomolecular imager (Cytiva) was used.

### Statistical and data analysis

Data analysis and all statistical analysis was performed using the R Statistical Software (version 4.0.4.). Sample size (n) for FRET experiments ranged from 12 to 35, from at least 3 independent experiments for each oligomer pair. Sample sizes of Ca^2+^ signal experiments are indicated in the figure legends.

### Structural models

Human SERCA/PLN homology model was built based on the rabbit complex (PDBID: 4kyt) and the human SERCA (PDBID: 7e7s) structures using Modeller^41^ (Fig. S3b and http://www.hegelab.org/resources.html). E protein was docked to the human SERCA model using PIPER/ClusPro^27^. The most suitable E protein pose (e.g. was not parallel with the membrane or did not show reverse membrane topology) was selected from the returned docked poses. AlphaFold2 was run locally with full database mode as described in^42,43^.

### Molecular dynamics simulations

The simulation systems were prepared using CHARMM-GUI^44,45^. First, the structural models of SERCA/PLN and SERCA/E protein were oriented according to the Orientations of Proteins in Membranes database^46^. Then N- and C-termini were patched with ACE (acetyl) and CT3 (N-Methylamide) groups, respectively. The membrane bilayer was symmetric containing 8:29:29:11:11:12 cholesterol:DMPC:POPC:DMPE:POPE:DMPI25 (dimyristoyl-phosphatidylcholine; 1-palmitoyl-2-oleoylphosphatidylcholine; dimyristoyl-phosphatidylethanolamine; 1-palmitoyl-2-oleoylphosphatidylethanolamine; dimyristoyl-inositol-4,5-bisphosphate). KCl was used at a concentration of 150 mM. Grid information for PME (Particle-Mesh Ewald) electrostatics was generated automatically, and the number of particles, pressure of 1 bar, and temperature of 310 K were constant. GROMACS 2022 with the CHARMM36m force field was used to run molecular dynamics simulations^47,48^. Each system was energy minimized using the steepest descent integrator, which stopped when the largest force in the system became less than 500 kJ/mol/nm. In order to increase sampling, several simulations (n = 3 for each system) were forked using the energy minimized system, with different velocities. Equilibration was performed in six steps and production runs were run for 500 ns. The corresponding parameter files are available for download. The trajectories were analyzed using MDAnalysis^49^ and NumPy. Molecular visualization was performed using PyMOL (Schrödinger, LLC). Graphs were generated using Python and its matplotlib library^50^.

## Supplementary Information

Supplementary Information.

## Data Availability

Our data are available at http://www.hegelab.org/resources.html or from the corresponding author (Tamás Hegedűs) upon request.
