# Supplementary material for: SARS-CoV-2 envelope protein alters calcium signaling via SERCA interactions
Source: Sci Rep. 2024 Sep 11;14:21200. doi: 10.1038/s41598-024-71144-5 (PMC11391011; doi:10.1038/s41598-024-71144-5)
Supplement: Supplementary file 1 — Supplementary Information. [file 41598_2024_71144_MOESM1_ESM.pdf]

Supplementary text

Heterogeneity of FRET values. In general, we recorded FRET values with a large variability. There were some cells in which no FRET was detected at all, and others in which particularly high FRET was observed. One reason for this in the case of regulins and E protein is their different subcellular localization (e.g. trafficking from ER to Golgi), so their intracellular distribution can be different from cell to cell. Interestingly, we observed quite different trafficking patterns for different regulins. For example, ALN seemed to reach the Golgi localization shortly after transfection. On the other hand, both regulins and E protein can homo-oligomerize with themselves or form hetero-oligomers with the others and the different oligomerization modes can compete with each other. Moreover, the amount of proteins in the cells and the proportion of proteins with the different fluorescence tags may also vary during co-expression.

FRET through a membrane bilayer. The physical interaction between E protein, regulins and SERCA were detected using AP-FRET with N-terminally labeled fluorescent constructs. The N-termini of both regulins and SERCA are cytosolic, while the E protein has opposite orientation with a luminal N-terminus (1). Since the C-terminally tagged E protein was not stable, we used N-terminal labels and detected energy transfer between the two fluorophores separated by a lipid bilayer. A similar setup was described previously for both plasma membrane (2,3) and endoplasmic reticulum membranes confirming the feasibility of our assay (4). While the ER membrane thickness can vary between 2.9-5.5 nm depending on the lipid composition (5), the fluorescence energy transferred from a donor fluorophore to an acceptor fluorophore is typically observed over distances between 1 nm and 10 nm (6). In addition, the E protein N-terminus is short, ending near the membrane/cytosol interface, thus it restrains the fluorescent protein close to the membrane. These observations support the validity of our FRET results.

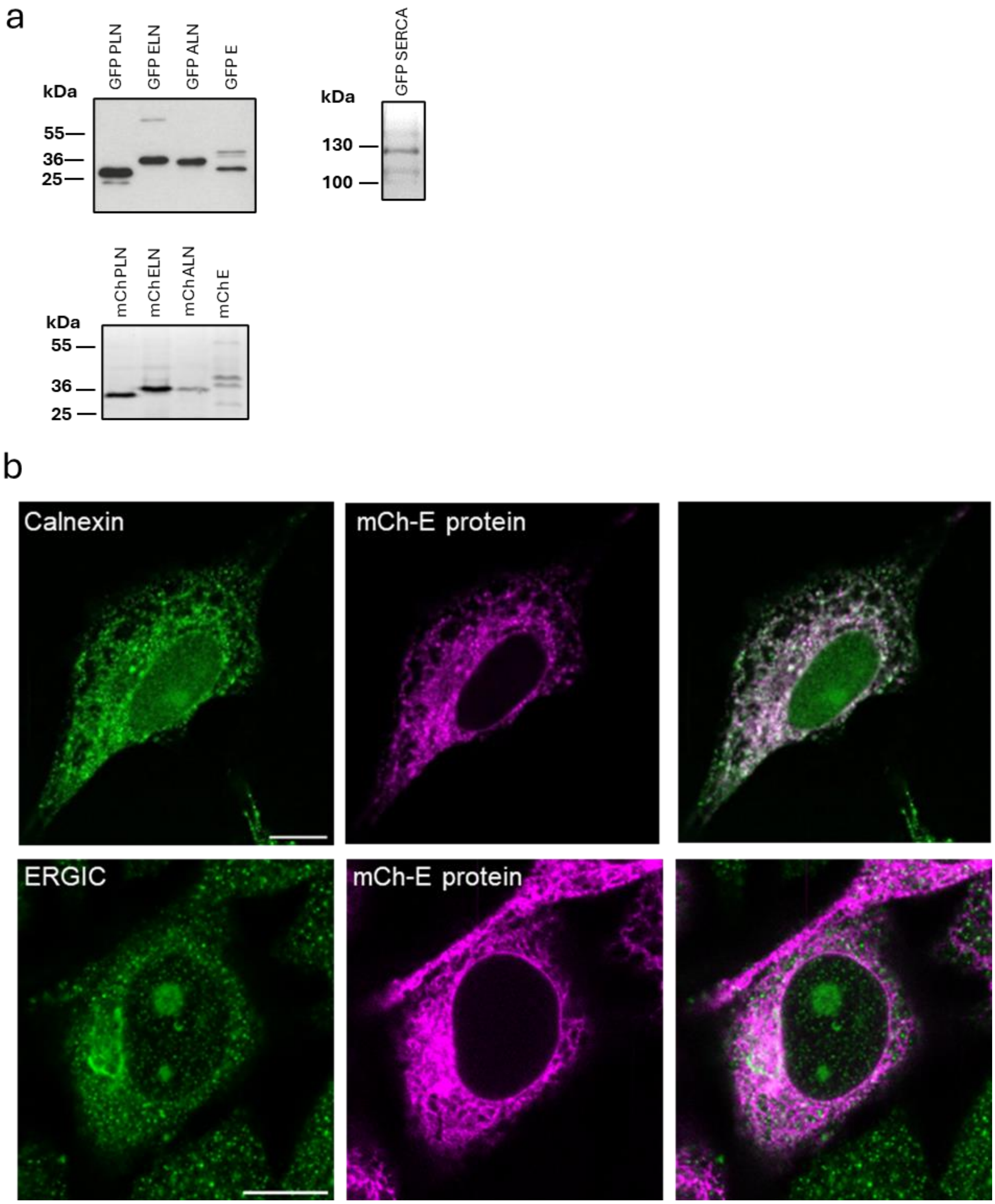

Figure S1. Expression and localization of regulins and E protein, related to Figure 2.
(**a**) The fusion protein constructs on Western blots with anti-GFP and anti-mCherry antibodies show the expected molecular weights. In some of the ALN and E protein constructs, the fluorescent protein was cleaved. As the cleaved fluorophore showed an easily recognizable diffuse cytosolic distribution, we were able to avoid these cells in our microscopy experiments.
(**b**) mCherry-E protein co-localizes with an ER marker (calnexin) and an ERGIC marker (α-ERGIC-53) in HeLa cells. In the first column ER and ERGIC markers are shown in green. Second column displays the distribution of mCherry-E protein. Third column exhibits the two channels together. Co-localized proteins are indicated by white color.

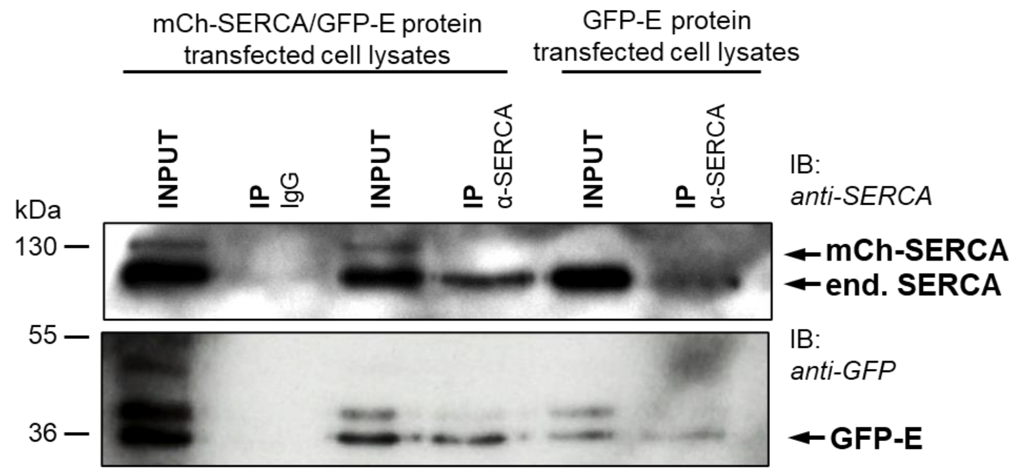

Figure S2. Co-immunoprecipitation experiment using anti-SERCA IID8 antibody confirmed an association between the eGFP-E protein and endogenous SERCA.

The first 4 lanes show the co-IP result obtained from Hela cells expressing mCherry-SERCA2b and eGFP-E protein. Western blot analysis was employed using anti-SERCA IID8 antibody and anti-GFP antibodies as indicated (IB). The negative control is presented by lanes 1 and 2, showing lysate (INPUT) and the eluted fractions (IP) using non-immune IgG1 in the co-IP assay. The lanes 3 and 4 show the lysate (INPUT) and the eluted fractions (IP) using anti-SERCA IID8 antibody. The lanes 5 and 6 show the co-IP result obtained from Hela cells expressing only eGFP-E protein. Lane 6 shows that the eGFP-E protein can also be pulled down by the endogenous SERCA protein.

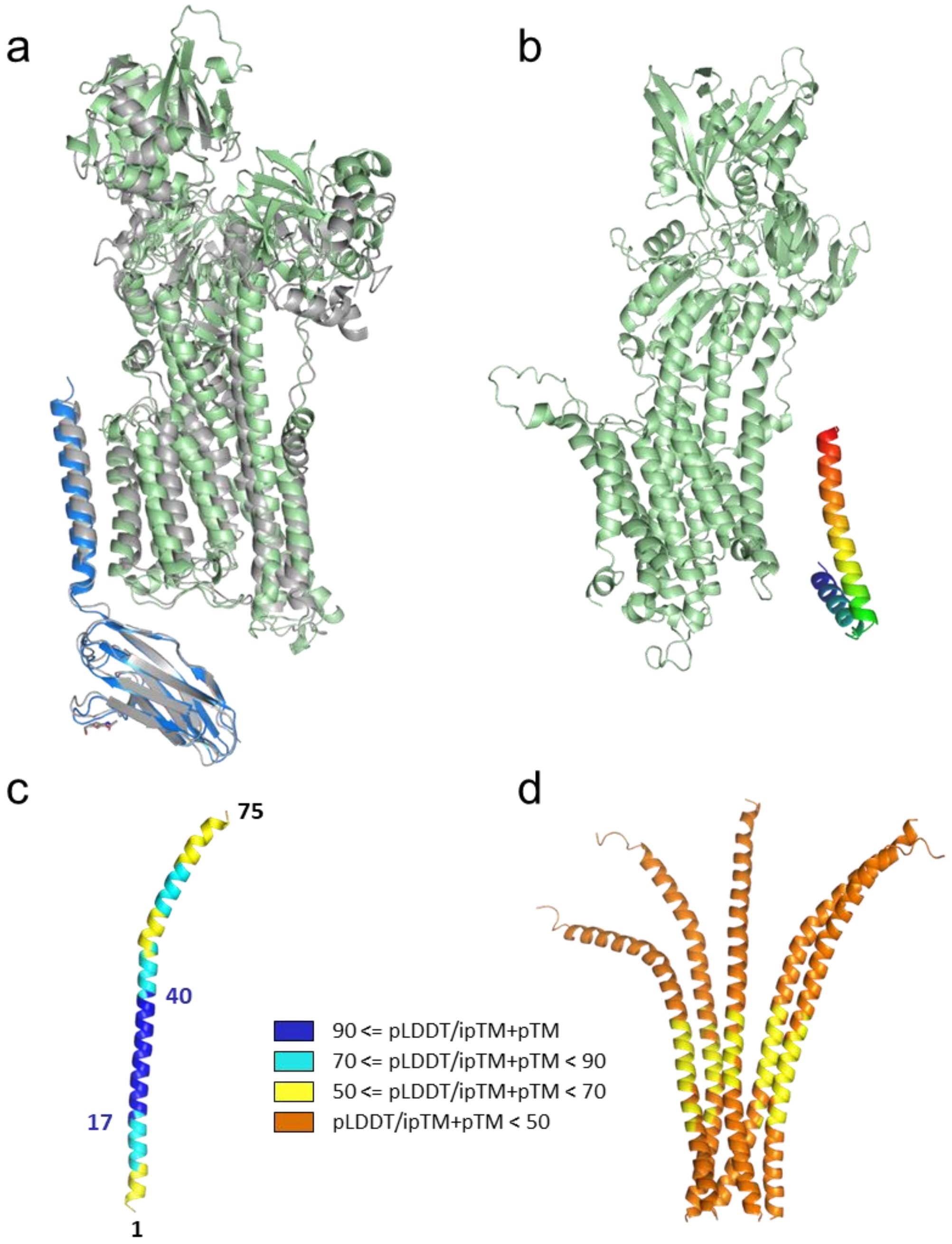

Figure S3. AlphaFold predictions of calcium pumps, regulins, and their complexes, related to Figure 5.

(**a**) AlphaFold correctly predicted the PMCA/neuroplastin complex (green and blue) when compared to its experimental structure (gray, PDBID: 6a69). (**b**) SERCA and PLN do not interact in the AlphaFold-predicted structure. The failure was likely caused by the low level of evolutionary information associated with this transient protein-protein interaction. (**c**) AlphaFold generated a model for the monomeric E protein, colored by pLDDT scores. (**d**) The pentameric AlphaFold model exhibited low quality as indicated by the low ipTM+pTM scores (color-coded).

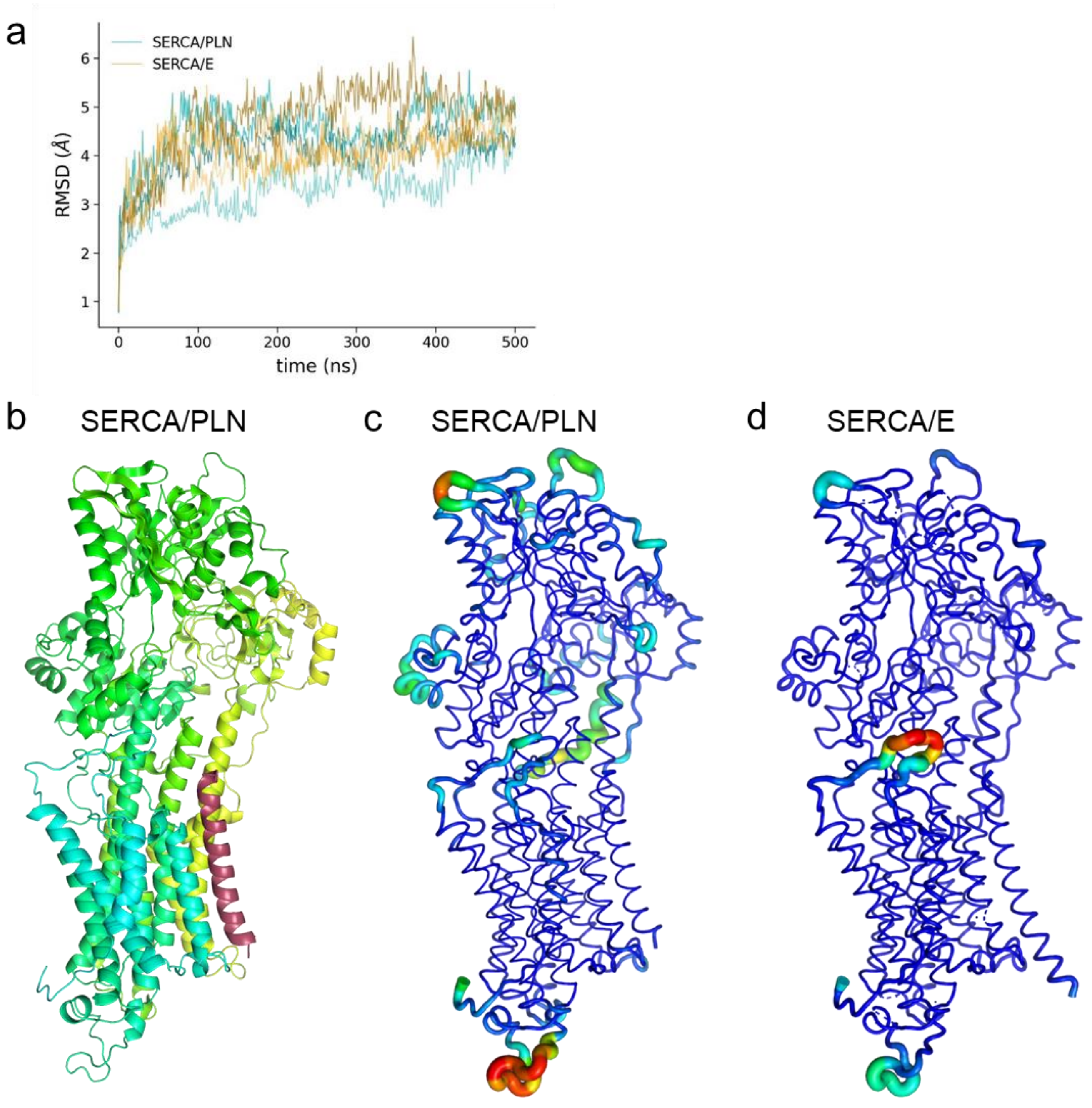

Figure S4. Dynamics of SERCA/PLN and SERCA/E complexes, related to Figure 5.
(**a**) RMSD of SERCA/PLN and SERCA/E complexes from the initial structure, calculated from 3-3 independent, 500-500 ns long trajectories. (**b**) Human SERCA/PLN homology model based on the rabbit complex (PDBID: 4kyt) and the human SERCA (PDBID: 7e7s) structures. SERCA is colored yellow-green-cyan and PLN is labeled by raspberry. (**c, d**) Structures in rainbow colors show the root mean square fluctuations calculated using GROMACS Tools and plotted using PyMOL. Warmer color and thicker representation indicate higher fluctuations.

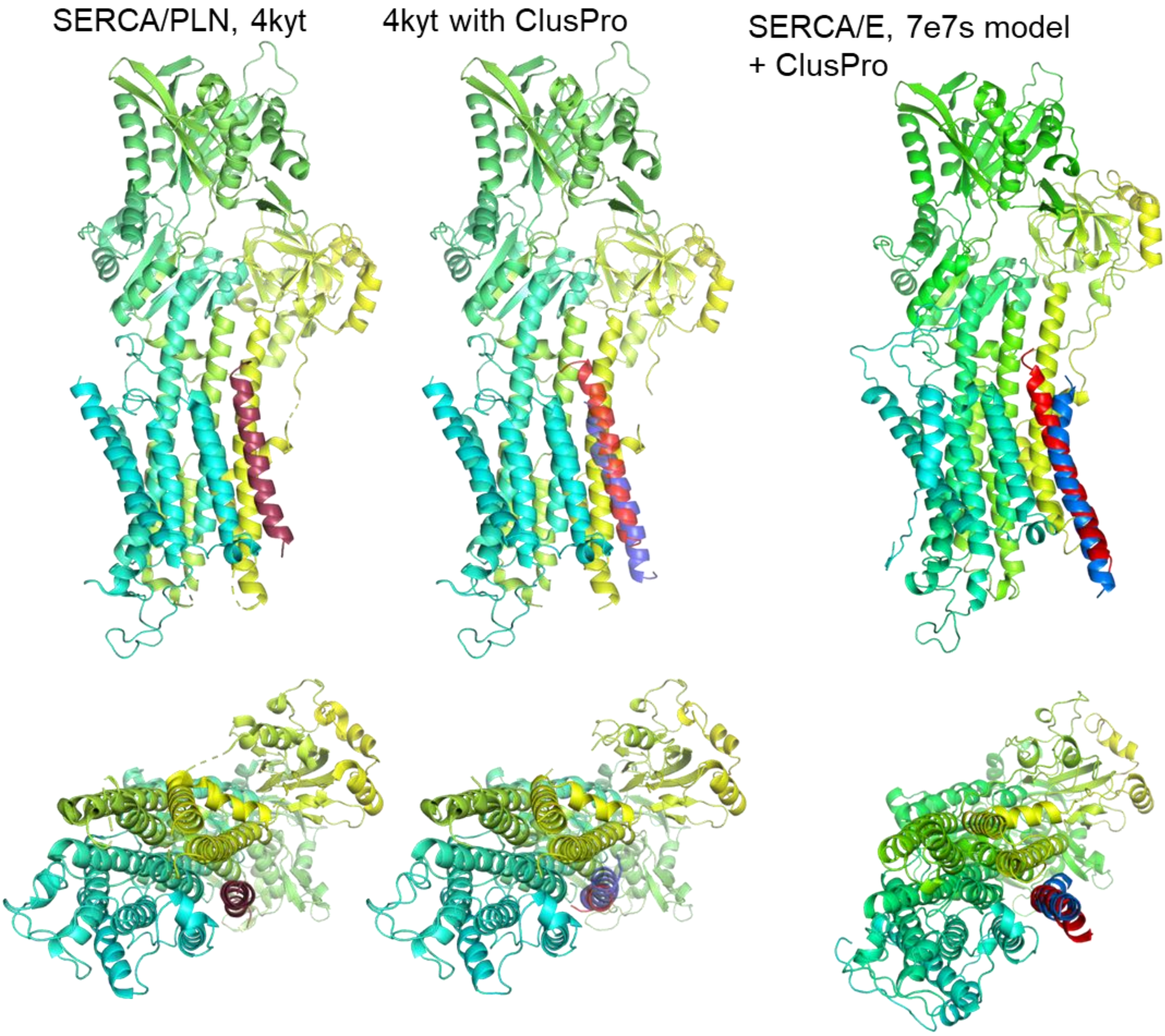

Figure S5. AlphaFold3 prediction of SERCA/E protein complex structure. The full-length SECA and E protein (residues X-Y) sequences were submitted for AlphaFold3 prediction (7). (**a**) The predicted SERCA structure exhibits a different conformation compared to the SERCA/PLN complex, with the E protein primarily binding to TM2 of SERCA. (**b**) Despite AlphaFold2's failure to accurately predict the SERCA/E protein complex, AlphaFold3 successfully produced a structure somewhat consistent with the SERCA/PLN complex. However, the reliability of this prediction remains low.

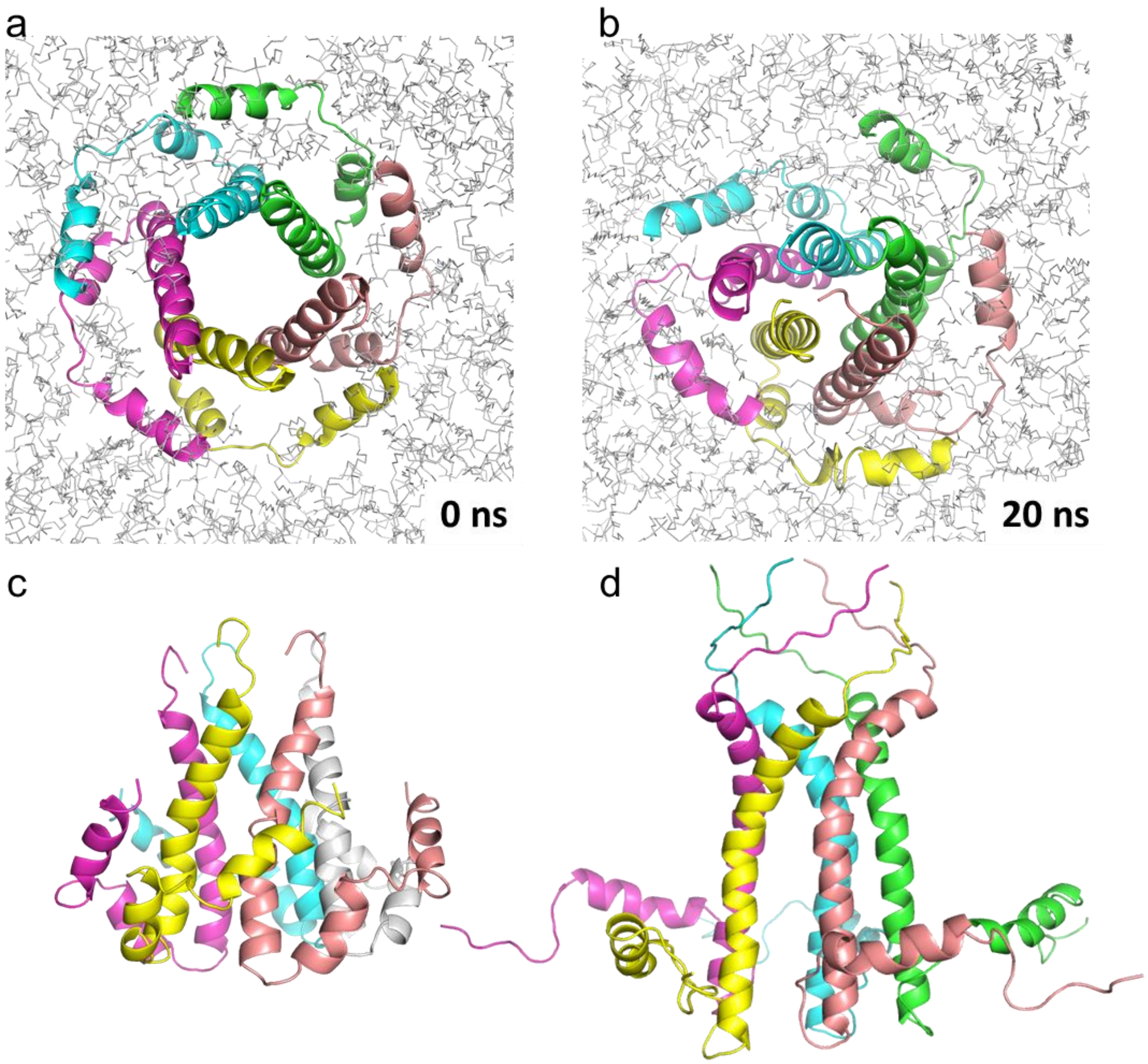

Figure S6. Structure and dynamics of E protein pentamers in MD simulations, related to Figure 7.

(**a, b**) E protein channel collapsed fast in MD simulations performed with structure from SARS-CoV-1 (PDBID: 5x29). (**c**) The NMR structure of this pentamer exhibited C-terminal regions immersed in the lipid bilayer. To exclude this conformation as a source of the collapse, we generated a simulation system only with TM regions (not shown), but it exhibited similar instability. (**d**) A pentamer was modeled based on a PLN homopentamer (PDBID: 2m3b), since the C-terminal helices in this structure were aligned in parallel with the membrane bilayer. However, the central channel was also not stable in this case and the luminal parts became highly bent.

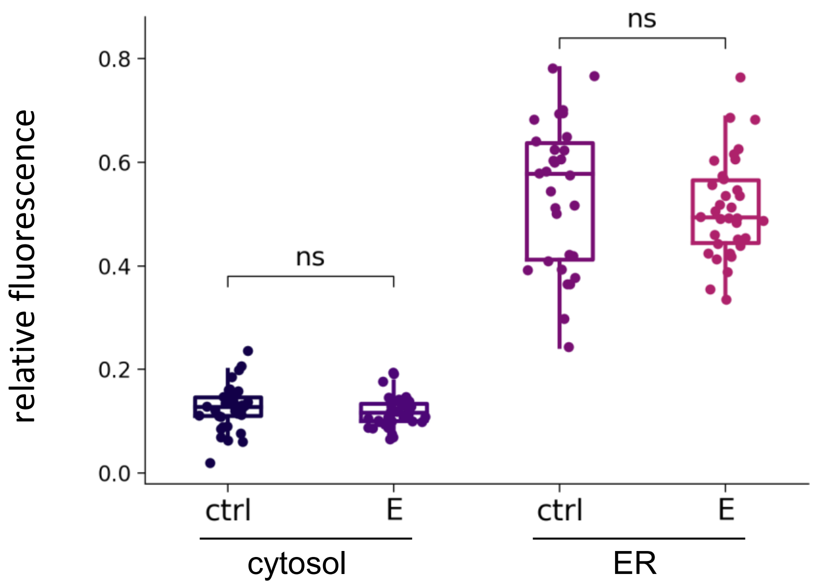

Figure S7. Expression of E protein does not significantly alter cytosolic and ER resting Ca2+ concentration.

Relative resting fluorescent signals of GCaMP2 cytosolic Ca^{2+} sensor (first two boxes) and ER-GCaMP-150 Ca^{2+} sensor (last two boxes) expressed alone (ctrl) or together with E protein (E) in HeLa cells. These results indicate the that resting gradient remained unchanged upon E protein expression. (ns: not significant; Kolmogorov-Smirnov test).

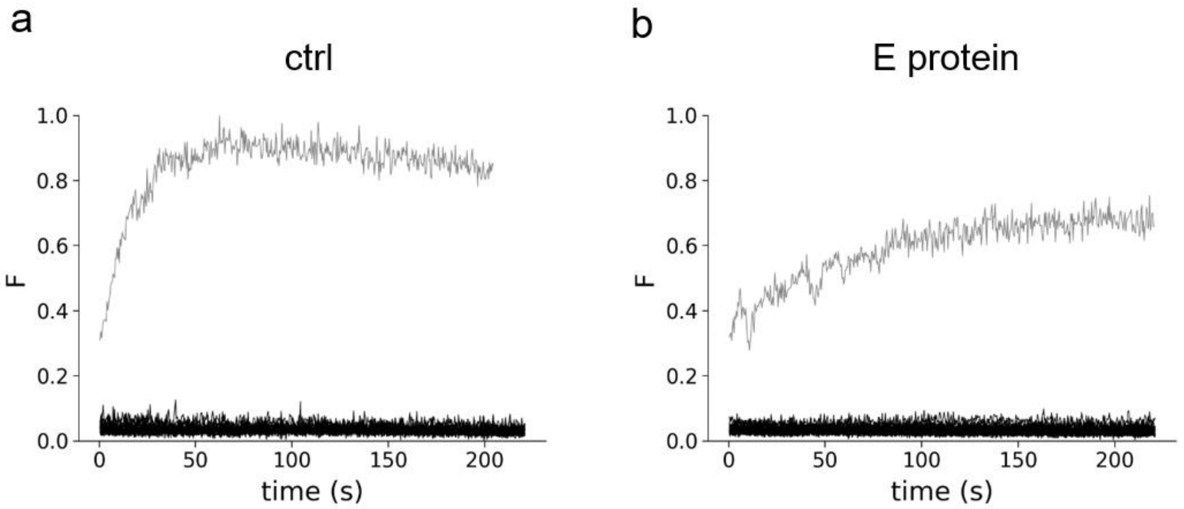

Figure S8. Inhibition of SERCA activity by thapsigargin completely abolished the ER reloading, related to Figure 8.

Experiments of Ca^{2+} reload into the ER were repeated with SERCA inhibitor thapsigargin (5 μM) in the absence (**a**) or in the presence of E protein (**b**). The plots show the relative fluorescence change of the ER-GCaMP6-150 Ca^{2+} sensor over time. Store depletion was achieved by ATP treatment in a calcium-free medium, and the ER Ca^{2+} concentration was monitored after adding Ca^{2+} back to the medium (black curves, n_{cntr}=17, $n_{Eprotein}$=24). An example curve for Ca^{2+} reload in the presence of active SERCA without inhibition is shown in gray from Figure 8.

**Western Blot**	**Bands considered**
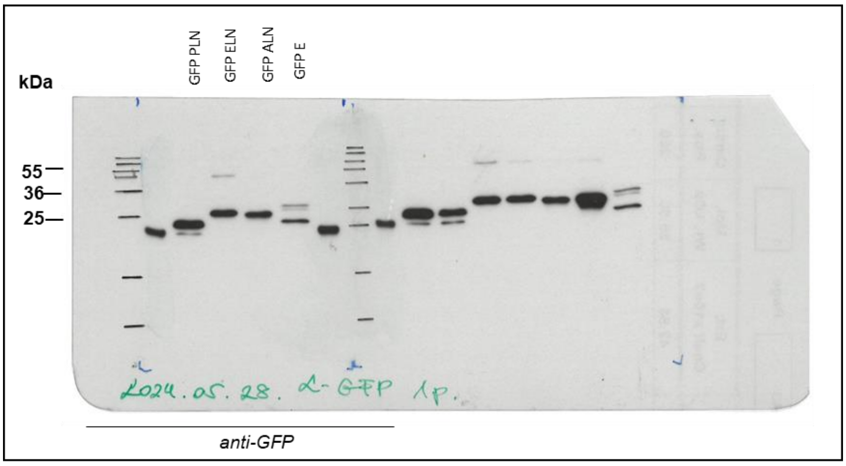<br><br>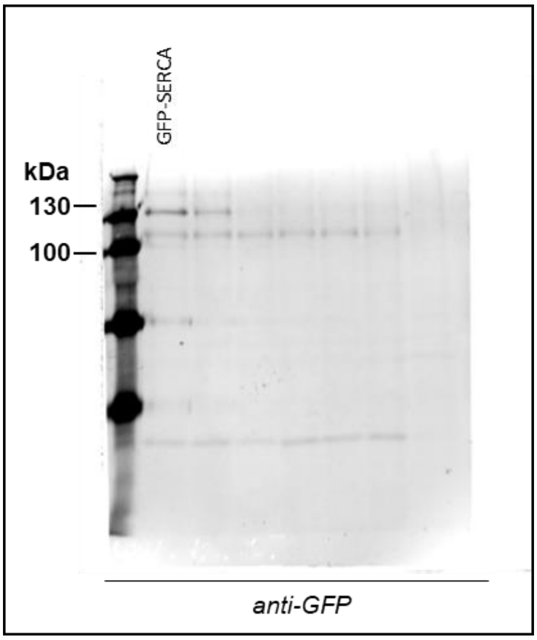 	GFP-tagged constructs displayed in Figure S1.<br><br>1st blot:<br>Anti-GFP antibody detects the full-length GFP-tagged regulins, GFP-E protein and their cleaved forms. The proteins were detected around 25 and 36 kDa, as expected. Samples were run in the same gel. Lanes shown in the main figure are labelled.<br><br>2nd blot:<br>Anti-GFP antibody detects the full-length GFP-SERCA. The protein was detected at 130 kDa, as excepted.<br>Lane shown in the main figure is labelled.

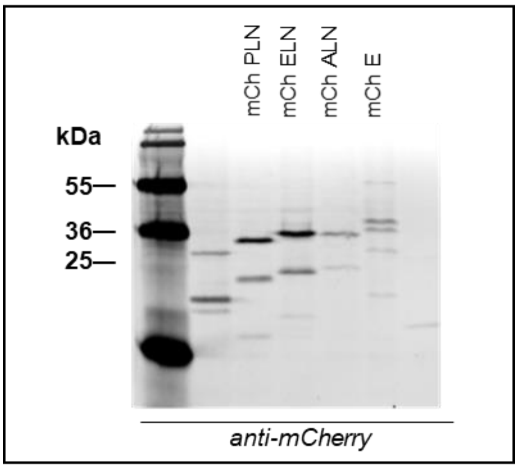

mCherry-tagged constructs displayed in Figure S1. Anti-mCherry antibody detects the full-length mCherry-tagged regulins, mCh-E protein and their cleaved forms. The proteins were detected around 25 and 36 kDa, as expected. Samples were run in the same gel. Lanes shown in the main figure are labelled.

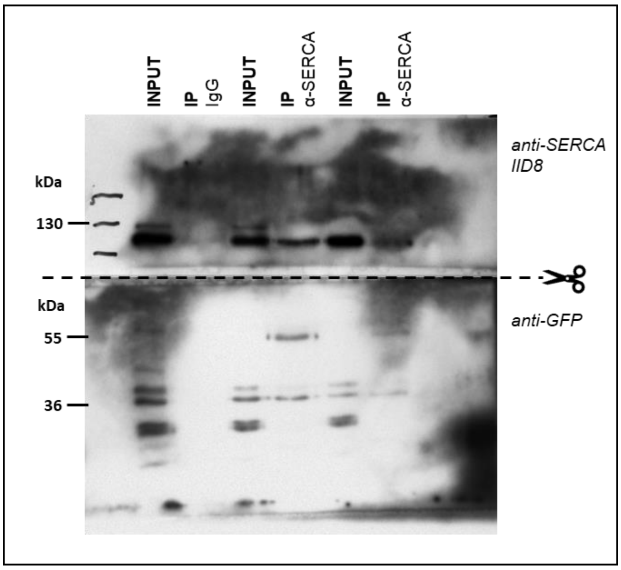

Co-IP results displayed in Figure S2. The original blot were cut in half before incubation with antibodies. The upper half with higher molecular weight proteins was probed with IID8 anti-SERCA antibody, while the lower half with smaller proteins with anti-GFP. GFP-SERCA was detected at 130 kDa and GFP-E protein was detected at 36 kDa, as expected. Lanes shown in the main figure are labelled.

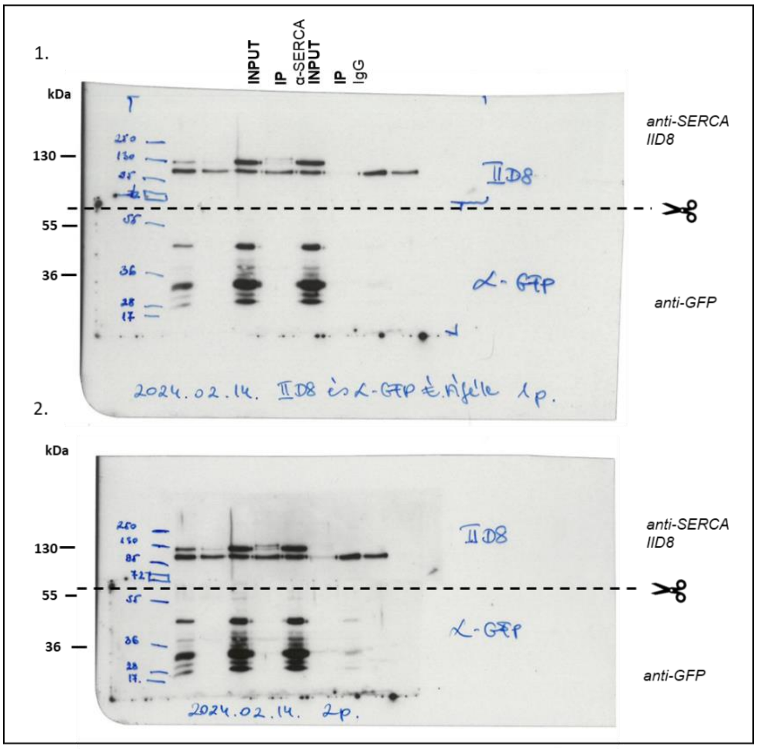

Co-IP results displayed in Figure 4. The original blot were cut in half before incubation with antibodies. The upper half with higher molecular weight proteins was probed with IID8 anti-SERCA antibody, while the lower half with smaller proteins with anti-GFP. GFP-tagged and endogenous SERCA was detected at 130 and 100 kDa and GFP-E protein was detected at 36 kDa, as expected. Lanes shown in the main figures are labelled.
1st blot: 1 min exposure time
2nd blot: 2 mins exposure time

Figure S9. Full length Western blots.
Full-length Western blots corresponding to the cropped blots of Figure S1a, Figure S2 and Figure 4. The blots indicate degradation of fluorescent-tagged proteins, the cleavage of GFP or mCherry tag, with a degree of degradation varying by transfection. In confocal microscopy, the cleavage of fluorophore is clearly visible as the cleaved fluorophore shows a diffuse cytosolic signal in contrast to the ER or ERGIC distribution of the full-length fluorescent-labeled proteins. In confocal experiments, these cells were excluded.

**Figure 3**				
**FRET / homo-oligomers**				Kolmogorov-Smirnov
	mean	SE		p value
ctrl	**0.991**	**0.651**	ctrl and E-E	**4.19E-08**
E-E	**19.648**	**2.521**	ctrl and PLN-PLN	**8.86E-09**
PLN-PLN	**18.289**	**2.159**	ctrl and ALN-ALN	**0.038**
ALN-ALN	**4.628**	**0.988**	ctrl and ELN-ELN	**0.00032**
ELN-ELN	**8.903**	**1.505**		

**Figure 4a**				
**FRET / hetero-oligomers**				Kolmogorov-Smirnov
**E - regulin, regulin-regulin**	mean	SEM		p value
ctrl	**0.991**	**0.651**	ctrl and PLN-E	**0.018680**
PLN-E	**5.448**	**0.916**	ctrl and ALN-E	**0.048760**
ALN-E	**5.678**	**1.788**	ctrl and ELN-E	**0.002913**
ELN-E	**5.587**	**0.914**	ctrl and PLN-ALN	**0.000465**
PLN-ALN	**7.111**	**1.047**	ctrl and PLN-ELN	**0.052690**
PLN-ELN	**3.477**	**0.692**	ctrl and ALN-ELN	**0.063150**
ALN-ELN	**3.446**	**0.710**		

**Figure 4b**				
**FRET / hetero-oligomers**				Kolmogorov-Smirnov
**SERCA-regulin**	mean	SEM		p value
ctrl	**0.991**	**0.651**	ctrl and SERCA-E	**0.013**
SERCA-E	**5.412**	**0.812**	ctrl and SERCA-PLN	**0.00018**
SERCA-PLN	**7.004**	**1.202**	ctrl and SERCA-ALN	**9.06E-07**
SERCA-ALN	**12.034**	**1.767**	ctrl and SERCA-ELN	**0.00093**
SERCA-ELN	**4.999**	**0.836**	ctrl and SERCA-PLN#	**0.00276**
SERCA-PLN#	**7.614**	**1.567**		

**Figure 6d**				
				Mann-Whitney-Wilcoxon
	mean	SEM		p value
ctrl pre	**0.539**	**0.026**	ctrl pre and E pre	**0.287**
ctrl post	**0.459**	**0.027**	ctrl pre and ctrl post	**0.0772**
E protein pre	**0.511**	**0.016**	ctrl post and E post	**0.000329**
E protein post	**0.299**	**0.029**	E pre and E post	**4.55E-08**

**Figure 7d**				
**Tg treatment**				Kolmogorov-Smirnov
	mean	SEM		p value
ctrl	**0.01123**	**0.00142**	ctrl and E	**0.154**
E protein	**0.00849**	**0.00087**		

**Figure 8d**				
**ER reload**				Kolmogorov-Smirnov
	mean	SEM		p value
ctrl	**0.04734**	**0.00255**	ctrl and E	**1.07E-10**
E protein	**0.02799**	**0.00537**		

**Figure 8g**				
**FWHM**				Mann-Whitney-Wilcoxon
	mean	SEM		p value
ctrl	**16.349**	**1.966**	ctrl and E	**0.00281**
E protein	**38.688**	**8.921**		

Table S1. Statistical parameters of the data presented in the figures.

References

1. Nieto-Torres JL, DeDiego ML, Álvarez E, Jiménez-Guardeño JM, Regla-Nava JA, Llorente M, et al. Subcellular location and topology of severe acute respiratory syndrome coronavirus envelope protein. Virology. 2011 Jul 5;415(2):69–82.

2. Sappakhaw K, Jantarug K, Slavoff SA, Israsena N, Uttamapinant C. A Genetic Code Expansion-Derived Molecular Beacon for the Detection of Intracellular Amyloid-β Peptide Generation. Angewandte Chemie International Edition. 2021;60(8):3934–9.

3. Haga Y, Ishii K, Hibino K, Sako Y, Ito Y, Taniguchi N, et al. Visualizing specific protein glycoforms by transmembrane fluorescence resonance energy transfer. Nat Commun. 2012 Jun 19;3(1):907.

4. Fernández-Dueñas V, Burgueño J, Ciruela F. Exploring Drug-Receptor Interaction Kinetics: Lessons from a Sigma-1 Receptor Transmembrane Biosensor. Frontiers in Pharmacology [Internet]. 2017 [cited 2023 Jun 9];8. Available from: https://www.frontiersin.org/articles/10.3389/fphar.2017.00004

5. Prasad R, Sliwa-Gonzalez A, Barral Y. Mapping bilayer thickness in the ER membrane. Sci Adv. 2020;6(46):eaba5130.

6. Algar WR, Hildebrandt N, Vogel SS, Medintz IL. FRET as a biomolecular research tool - understanding its potential while avoiding pitfalls. Nat Methods. 2019 Sep;16(9):815–29.

7. Abramson J et al. Accurate structure prediction of biomolecular interactions with AlphaFold 3. Nature. 2024 Jun;630(8016):493-500.
